# Dynamic restructuring of supported metal nanoparticles and its implications for structure insensitive catalysis

**DOI:** 10.1038/s41467-021-27474-3

**Published:** 2021-12-07

**Authors:** Charlotte Vogt, Florian Meirer, Matteo Monai, Esther Groeneveld, Davide Ferri, Rutger A. van Santen, Maarten Nachtegaal, Raymond R. Unocic, Anatoly I. Frenkel, Bert M. Weckhuysen

**Affiliations:** 1 Inorganic Chemistry and Catalysis group, Debye Institute for Nanomaterials Science, Utrecht University, Universiteitsweg 99, 3584 CG Utrecht, The Netherlands; 2 Schulich Faculty of Chemistry, Technion - Israel Institute of Technology, 3200003 Haifa, Israel; 3 BASF Nederland B.V., Strijkviertel 61, 3454 PK De Meern, The Netherlands; 4 Paul Scherrer Institute (PSI), 5232 Villigen PSI, Switzerland; 5 Schuit Institute of Catalysis, Laboratory of Inorganic Chemistry and Catalysis, Eindhoven University of Technology, 5600 MB Eindhoven, The Netherlands; 6 Center for Nanophase Materials Sciences, Oak Ridge National Laboratory, Oak Ridge, TN USA; 7 Department of Materials Science and Chemical Engineering, Stony Brook University, Stony Brook, NY USA; 8 Division of Chemistry, Brookhaven National Laboratory, Upton, NY 11973 USA

**Keywords:** Heterogeneous catalysis, Catalytic mechanisms, Nanoparticles

## Abstract

Some fundamental concepts of catalysis are not fully explained but are of paramount importance for the development of improved catalysts. An example is the concept of structure insensitive reactions, where surface-normalized activity does not change with catalyst metal particle size. Here we explore this concept and its relation to surface reconstruction on a set of silica-supported Ni metal nanoparticles (mean particle sizes 1–6 nm) by spectroscopically discerning a structure sensitive (CO_2_ hydrogenation) from a structure insensitive (ethene hydrogenation) reaction. Using state-of-the-art techniques, *inter alia* in-situ STEM, and quick-X-ray absorption spectroscopy with sub-second time resolution, we have observed particle-size-dependent effects like restructuring which increases with increasing particle size, and faster restructuring for larger particle sizes during ethene hydrogenation while for CO_2_ no such restructuring effects were observed. Furthermore, a degree of restructuring is irreversible, and we also show that the rate of carbon diffusion on, and into nanoparticles increases with particle size. We finally show that these particle size-dependent effects induced by ethene hydrogenation, can make a structure sensitive reaction (CO_2_ hydrogenation), structure insensitive. We thus postulate that structure insensitive reactions are actually *apparently* structure insensitive, which changes our fundamental understanding of the empirical observation of structure insensitivity.

## Abstract (web summary)

Structure insensitivity in catalysis has been empirically observed, but no satisfactory theoretical explanation could be given. By studying different nanoparticle sizes under dynamic catalytic conditions reaction-dependent particle size dependent restructuring was linked to the aforementioned.

## Introduction

Supported metal nanoparticles are a highly important class of heterogeneous catalysts, used for a range of applications: from food preparation to bulk and specialty chemical synthesis as well as emissions control. Studying nanoparticular systems under catalytic reaction conditions is far from trivial, spectroscopic signals are generally very low, for surface sensitive spectroscopies, and bulk techniques. Firstly so, because the fraction of exposed surface on a nanoparticle is low^1,2^. Of that small fraction of surface, an even smaller fraction is active. There is generally a low signal-to-noise ratio^3^. Secondly, the variety of adsorption sites is large and ill-defined, and there are multiple surface elementary processes happening in tandem. There are broad and convoluted spectroscopic signals^4–6^. Thirdly, relevant surface processes consist of several consecutive elementary reaction steps. The adsorbate-surface systems change dynamically^7,8^, which is system-inherent and thus desirable to study. Experimentally this is highly challenging, particularly so considering reasons 1 and 2 listed above. As such, an overwhelming majority of studies in literature have simplified the systems under study for example by using single crystal facets, studied under ultra-high vacuum conditions (an approach that is termed surface science)^8–13^. These approaches have yielded many of the important insights that we currently build our understanding on. However, in recent years it has also become apparent that adsorbates and surfaces have completely different physical parameters, such as surface stability, mobility of species, surface coverages, and surface energies, at relevant conditions of pressure and temperature^12,14,15^.

Structure (in)sensitivity is a fundamental physical concept in catalysis, which relates the rate of a catalytic reaction per unit surface area to the size of a nanoparticle^16^. If this rate per unit surface area changes with catalyst particle size, a reaction is termed structure sensitive. Conversely if it does not—a reaction is termed structure insensitive. For structure sensitivity, surface science studies have unequivocally shown that in order for the cleavage or formation of certain bonds to proceed efficiently, some specific groupings of surface atoms are required^17–21^. The population of these surface sites varies with nanoparticle size. It is commonly accepted to classify reactions as being either of two types of structure-sensitive, or to be structure-insensitive depending on whether, and how the surface-specific rate (or turnover frequency, TOF) is a function of particle size. Structure insensitive reactions empirically show no - or very little- dependence of reaction rate on particle size. A typical example is the hydrogenation of alkenes^20^. Despite the fact that not many heterogeneous catalytic reactions have been studied more intensively than olefin hydrogenation^22–24^, these classic examples of structure insensitive reactions are still not entirely understood. It has been noted^8,16,21^ that it is surprising that the rate of *any* reaction should be *insensitive* to the different sites exposed on different sizes of nanoparticles. Different explanations have been given for the observed effects, from carbonaceous overlayers^25^, to a very strong structural dependence on a specific surface site^21^ (it must be noted that this explanation obviously only holds for single crystal facets in surface science), to surface restructuring^8^. All of these explanations stem from surface science studies (though restructuring has been shown to occur under reaction conditions^26,27^), yet single crystal facets studied under vacuum conditions are in stark contrast to the supported nanoparticles in practical catalyst materials, which are structurally much more dynamic, and inhomogeneous. To enhance our fundamental understanding of such principles as structure sensitivity on working catalysts, it is of the essence to develop and apply time-resolved analytical techniques^12,28^ to simultaneously monitor the dynamic changes that occur on *both* sides of chemical bond formation on the surface of a solid catalyst: the adsorbate (often organic) and the substrate (often inorganic).

Here we study how the size of metal catalyst particles affects dynamic surface changes during structure sensitive, and “structure insensitive” catalysis with operando millisecond quick-X-ray absorption spectroscopy (quick-XAS), operando Fourier Transform infrared spectroscopy (FT-IR) both with on-line product analysis via an ultra-fast GC, and in-situ high resolution transmission electron microscopy (HR-TEM). A deeper understanding of such fundamental concepts in the interface chemistry of nanoparticles under working conditions can ultimately help us to rationally influence reaction rates by intelligent control of active sites.

## Results and discussion

### Counterintuitive X-ray absorption spectroscopy results

It is clear that much is still not understood with respect to the concept of structure insensitivity in catalysis. To this end, here we will examine the nature of the behavior of a set of SiO_2_-supported Ni nanoparticular catalysts with mean particle sizes ranging from 1–6 nm, under working conditions. The catalysts are compared in a classically structure sensitive reaction (CO_2_ hydrogenation), and structure insensitive (ethene hydrogenation) reaction with state-of-the-art operando spectroscopies in an attempt to understand dynamic changes onset by catalysis on nanoparticles. Three powerful characterization techniques are used to this end, two bulk techniques; operando Fourier transform infrared spectroscopy, operando quick-X-ray absorption spectroscopy, and a single particle technique; in-situ high resolution transmission electron microscopy.

**Fig. 1:**
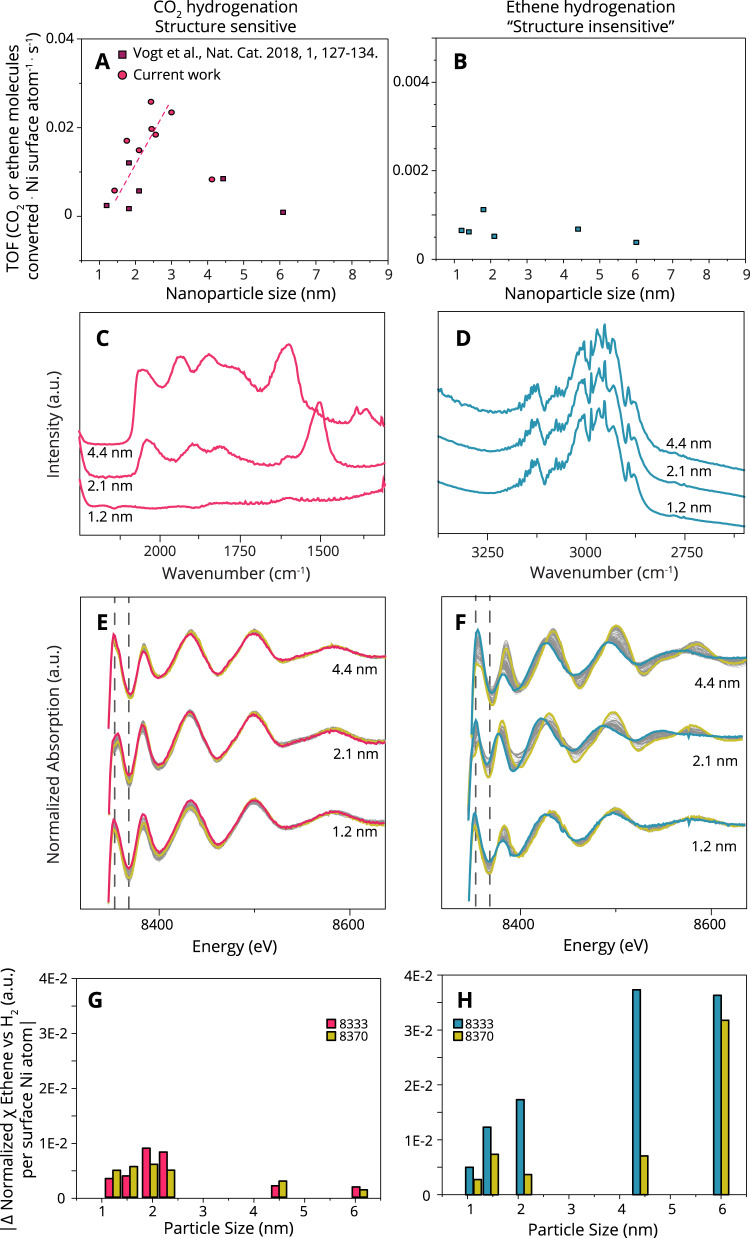
Structure sensitivity versus structure insensitivity as observed by different spectroscopic techniques. **a**, **b** Structure sensitivity versus structure insensitivity plotted on a linear Y-axis. The influence of the Ni mean particle size on TOF for **a** CO_2_ hydrogenation at 300 °C and **b** ethene hydrogenation at 150 °C. The Supplementary Information S1 shows alternative plots, and an extensive literature search on both structure sensitive, and insensitive reactions. **c**, **d** Operando FT-IR spectra during CO_2_ hydrogenation at 300 °C and ethene hydrogenation at 150 °C, respectively for three different mean particle sizes of Ni/SiO_2_. X-ray absorption spectra (XAS) with 2 s time resolution obtained upon switching from pure H_2_ after reduction, to either CO_2_ (**e**) or ethene (**f**) hydrogenation. **g**, **h** absolute magnitude of change induced by the onset of catalysis (seen in **e**, **f**) at the Ni K-edge using the largest Δ, at 8333 eV and the second largest Δ at 8370 eV indicated by the dotted lines in (**e**, **f**) and normalized for available surface area, for CO_2_ (**g**) and ethene (**h**) hydrogenation.

The catalysts with different mean particle sizes were first employed in catalytic testing in these reactions to verify their respective structure (in)sensitivity. Figure 1a, b show the particle size dependent TOFs plotted on a linear scale. Thereby we can deduce that for CO_2_ hydrogenation the activity shows a significant change with particle size (showing both new data, and data from a previous publication^29^), while for ethene hydrogenation such a change in activity is much less apparent. Supplementary Note 1 of the Supporting information alludes to the full catalytic details of these reactions, including an extensive literature review on structure sensitive versus reactions that are apparently structure insensitive, which ends by comparing our catalytic results to the available literature on the structure (in)sensitivity of these reactions. Supplementary Methods in the Supporting information gives additional information on the applied materials and methods.

Figure 1 shows operando spectroscopic (FT-IR, and XAS) data for the structure sensitive CO_2_ hydrogenation, and the “structure insensitive” ethene hydrogenation reactions. The vibrational frequencies in the IR spectra for the reaction intermediates of structure sensitive CO_2_ hydrogenation differ greatly with different mean Ni particle size (Fig. 1c). Most notable is the region for top- and bridge-adsorbed CO (2060–1900 cm^−1^), and in the formate region (~1590 cm^−1^). This has been noted before and has been found to explain the structure sensitivity of this reaction, as an optimal binding strength of CO is required^29^. On the other hand, for the structure insensitive ethene hydrogenation (Fig. 1d) the vibrational frequencies in the IR spectra for different particle sizes show no differences for different particle sizes. The main species that are detected for ethene hydrogenation (H_2_:ethene 1:1) are CH_3_ stretch vibrations of gaseous ethane at 2980 cm^−1^ and CH_2_ stretch vibrations of chemisorbed ethene at 2893 cm^−1^ (refs. ^30–32^). Supplementary Fig. 14 shows that at high ratios of H_2_:ethene, different intermediates are in fact detectable (for example the C-H stretch from methane at 3015 cm^−1^)^33^. Thus, the different mean Ni particle sizes in Fig. 1b truly share very similar intermediates during ethene hydrogenation.

While FT-IR is a powerful vibrational spectroscopic tool to detect organic species, reactants, products and intermediates of heterogeneous catalytic reactions, operando hard X-ray absorption spectroscopy (XAS, Fig. 1e, f), is a powerful tool to study the inorganic aspects of a catalyst. For example, changes in the local electronic structure which could appear through the introduction of adsorbates or by the onset of a catalytic reaction (the ‘Supplementary Methods’ section, and ‘Supplementary Discussion – X-ray Absorption Spectroscopy’, more specifically Supplementary Figs. 15–S22 give additional details on our XAS measurements). The local electronic structure of Ni is probed in this case by measuring the K-edge at 8333 eV. When XAS spectra are normalized, a change in the features of the X-ray absorption near edge structure (XANES) indicates a quantifiable change in electronic structure and/or geometry of our Ni catalyst nanoparticles. In Fig. 1e, f, operando XANES spectra are shown for 3 samples with differing mean Ni particle sizes, recorded during a switch from H_2_ to catalysis - ethene, or CO_2_ hydrogenation (1:1 ethene:H_2_, 1:4 CO_2_:H_2_).

Interestingly, when comparing the XANES spectra for CO_2_ hydrogenation (Fig. 1e) and ethene hydrogenation (Fig. 1f), there is a clear difference comparing the two reactions. This is for example seen by the shift in energy of the features around 8390 and 8415 eV, indicating that the local electronic change that is induced by ethene hydrogenation (the structure insensitive reaction) is much larger than the electronic change onset by CO_2_ hydrogenation (the structure sensitive reaction). Hence, while FT-IR aligns well with expectations based on activity for the structure sensitive and insensitive reactions (Fig. 1a, b), the corresponding changes observed with XAS seem counterintuitive at first glance. Cargnello et al. recently showed that the correlation of certain particle size parameters with activity trends is an excellent way to determine the cause of activity trends^34^. Along this line of thought, the fact that these two parameters do not align requires further investigation.

Figure 1g, h shows the two largest absolute changes in X-ray absorption (identified at X-ray energies 8333 and 8370 eV, indicated in Fig. 1e, f by dotted gray lines). These figures show in a more quantitative way that the changes in the XANES are larger for ethene hydrogenation than for CO_2_ hydrogenation. These values were normalized to the exposed surface area. If one compares the overall magnitude of changes onset by CO_2_ hydrogenation, versus those that are onset by ethene hydrogenation, it is clear that in the latter case more restructuring occurs for all particle sizes. Interestingly, the particle size dependence of the restructuring shows that the most active mean particle size for CO_2_ hydrogenation also restructures the most (Fig. 1a, g). For ethene hydrogenation, the degree of restructuring increases with increasing Ni NPs size (Fig. 1h). Interestingly, larger Ni particles are perturbed much more than smaller ones even after normalization to the amount of exposed surface. It is remarkable to see that the degree of restructuring for ethene hydrogenation is highly particle size dependent but the TOF (Fig. 1b) is not.

### Sub-second EXAFS reveals size-dependent restructuring trends

**Fig. 2:**
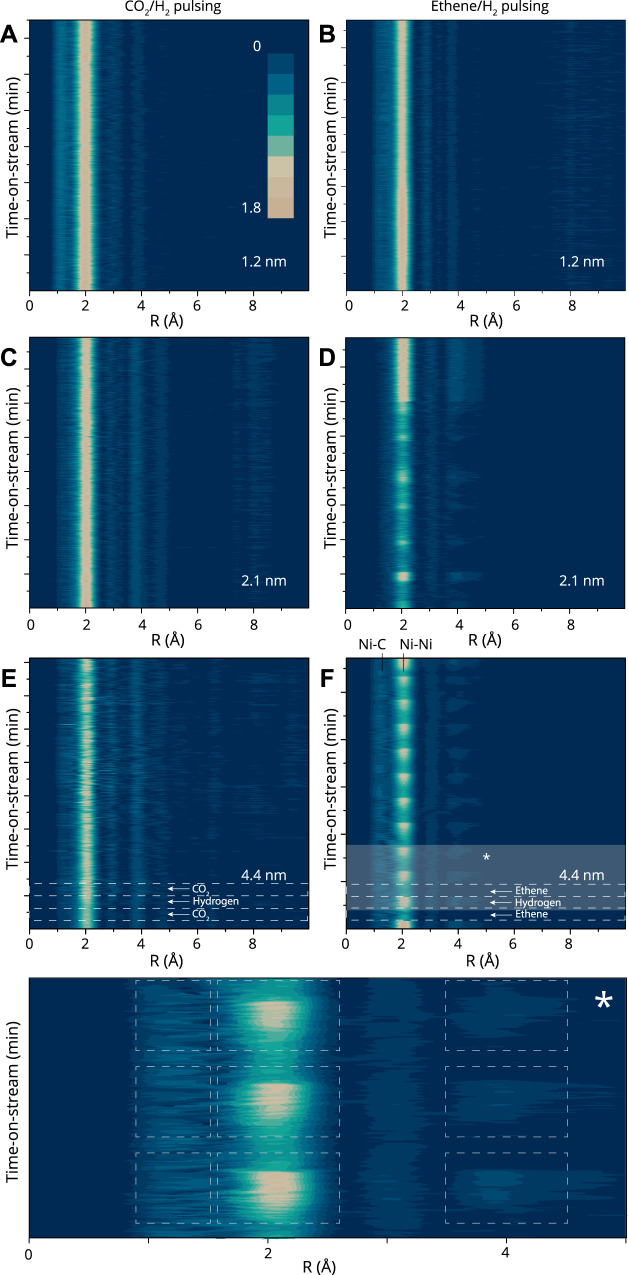
Operando time-resolved quick-EXAFS during feedstock pulses shows particle-size-dependent restructuring. Top view of the Fourier transform of the quick-XAS spectra at 2 s time resolution of feedstock pulsed experiments for 3 catalyst samples with different mean Ni particle sizes. See also relevant EXAFS in the Supporting information, Supplementary Figs. 15 and 28. **a**, **c**, **e** pulsing CO_2_ and H_2_ at 400 °C and **b**, **d**, **f** pulsing ethene and H_2_ at 150 °C and ambient pressure. See Supplementary Fig. 29 for the average EXAFS of each experiment shown in **a**–**f**.

By studying the dynamic nature of the perturbation of the metal nanoparticles under catalytic conditions, we can obtain additional insight into the interesting observation that the structure insensitive reaction shows particle-size dependent restructuring. To the best of our knowledge, no such experimental study has been done on the particle size dependent nature of restructuring in catalysis^35^. To obtain time resolved information, we measured quick-X-ray absorption spectra with 100 ms time resolution (before binning) during alternating pulses of CO_2_ or ethene, alternated by pure hydrogen (see Supplementary Information section ‘Supplementary Methods’, and Supplementary Figs. 15–22). Similar trends were observed for catalytic conditions (that is, pulses of H_2_ alternated with H_2_ + reactant, Supplementary Fig. 21). All the spectra were recorded while measuring the products by gas chromatography (GC) with <10 s time resolution to ensure working catalytic conditions (see also Supplementary Fig. 22). The Fourier-transformed extended X-ray absorption fine structure (EXAFS) for the CO_2_ experiment alongside the ethene experiment are plotted in Fig. 2, binned to 2 s time resolution to increase the signal to noise ratio. The spectra are plotted sequentially in time on the Y-axis, with the intensity expressed by the color scale (see also Supplementary Figs. 24–29, and in particular Supplementary Fig. 29 for the average EXAFS of each experiment shown in Fig. 2a–f).

Notably, the Ni-Ni peak at approximately 2 Å (uncorrected for the photoelectron phase shift) increases systematically during ethene pulses by approximately 0.05 Å (e.g., in Fig. 2f), while no such periodic changes are observed for CO_2_ pulses. This observation indicates that the average Ni-Ni bond expands under ethene and, subsequently, contracts under H_2_. A second remarkable feature, visible in Fig. 2d and again most notably in Fig. 2f, is that the degree of local disorder in the catalyst sample changes reversibly during pulses of ethene and H_2._ This can be seen by the (dis)appearance of Ni-Ni multiple scattering paths at around 4 Å, which decrease (under ethene) and increase (under hydrogen) in intensity with a constant, and low noise level. Furthermore, a shoulder appears at 1.50 Å, in concert with the disordering of the particle. This can more clearly be seen in Supplementary Fig. 15. On the basis of the visual observation of these EXAFS data in Fig. 2 we can hypothesize that increased Ni-Ni bond length disorder is caused by the intercalation of carbon through the surface layers of Ni nanoparticles. Most interestingly, a particle-size dependent degree of reversibility of these structural changes^35^, correlating with ethene pulses, can also be seen in Fig. 2f. The reversibility of this expansion and contraction, or “breathing”, is particle size-dependent as can be seen in Fig. 2d where for the 2.1 nm catalyst sample that is shown, this “breathing” appears to halt after a few pulses, while it is not visible for the smallest particle size.

We can finally confirm this qualitative picture by quantitative EXAFS data analysis, as described in detail below (see also section ‘Supplementary Discussion – X-ray Absorption Spectroscopy’ in the Supporting information). By using the fits (FEFF6) of the time-resolved EXAFS data, we determined the variation of σ^2^ (the mean-square relative displacement, MSRD) in the EXAFS equation, which can be interpreted as a measure of distortion for a sample at a given temperature. Because the temperature was constant as measured by a thermocouple inserted in the catalyst bed (and repetitions at lower temperatures yield similar trends, see Supplementary Fig. 20) we conclude that the nature of the variation in MSRD is configurational, and not due to vibrational disorder. If caused by the diffusion of carbon through the outer layers of Ni nanoparticles, the time-derivative of the MSRD data should be comparable - by dimensionality argument at least - to the diffusion coefficient of carbon in nickel as we discuss in more detail below.

**Fig. 3:**
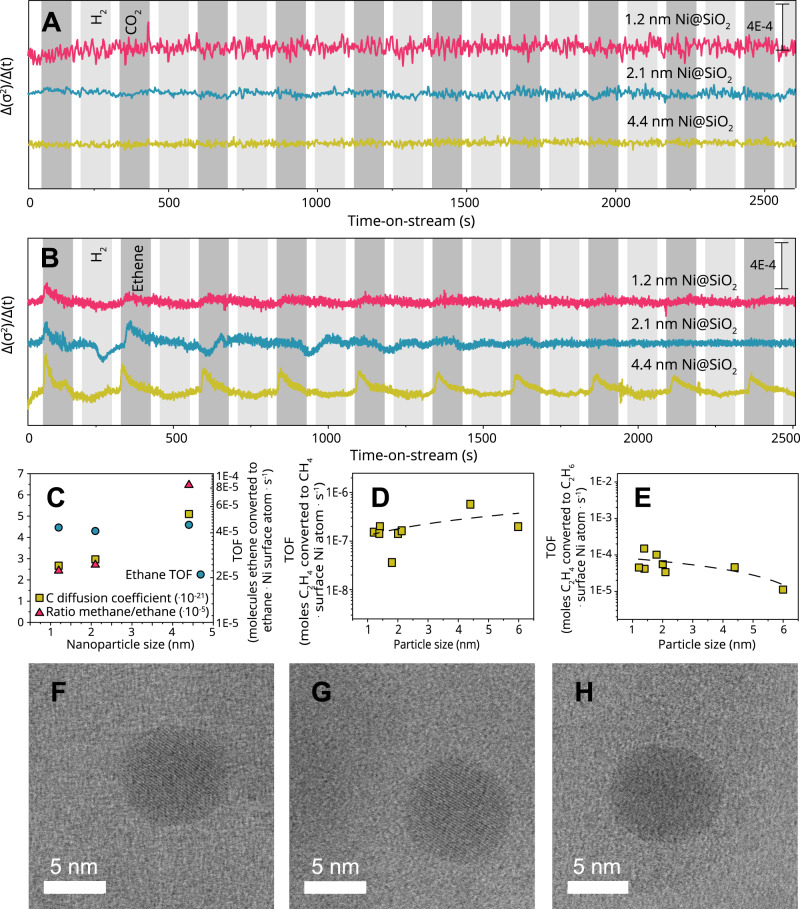
Dynamics and extent of adsorbate-mediate restructuring under ethene flow, and its impact on products distribution. **a**, **b** Batch analysis of CO_2_- and ethene-pulsed modulated excitation experiments shown in Fig. 2. For each particle size, 50.000–70.000 spectra were individually fit, fixing the coordination number but varying σ^2^. The values have been given an arbitrary offset for clarity. **c** The diffusion coefficient of C as calculated from time-resolved XAS measurements, plotted with the ethane TOF and the ratio of methane/ethane for 3 catalyst samples with different mean Ni particle sizes. **d**, **e** The turnover frequency (TOF) of ethene converted to methane (**d**) and ethane (**e**) per surface nickel atom per second, measured at 150 °C 1:1 H_2_:ethene with a GHSV of 2.000. **f**, **g**, **h** In-situ BF-STEM images of nanoparticles deposited directly onto SiN membrane via spark ablation, in gaseous environment of **f** H_2_ at 150 °C, **g** ethene at 150 °C, **h** and H_2_ at 150 °C.

Supplementary Fig. 26 shows σ^2^ plotted against time on stream. Figure 3a, b show the derivative ($d{\sigma }^{2}/{dt}$) plotted against time on stream during the pulsing experiments of CO_2_ and ethene, respectively, which are shown in Fig. 2. As emphasized above, due to the isothermal conditions of the gas switching experiments, the behavior of the derivative over time of σ^2^ as plotted in Fig. 3a, b should be interpreted as primarily caused by restructuring that is, evidently, adsorbate-induced. Thus, with the extraction of Δ_t_σ^2^ from the time-resolved quick-XAS data, we now have a parameter displaying the magnitude of restructuring of the particle, which allows us to deduce kinetic information of this observed restructuring for the catalyst samples with differing mean Ni nanoparticle size. This is highly interesting, because we observed that the structure insensitive reaction restructures supported metal nanoparticle catalysts *particle size-dependently*.

Figure 3b shows Δ_t_σ^2^ for structure insensitive ethene hydrogenation, from which we can note some very interesting trends. Firstly, Fig. 3b shows that the reversibility of ethene-mediated restructuring decreases with decreasing catalyst particle size. That is, the sample with larger mean Ni particle size shows changes of features in time that continue to correspond to the applied external gas throughout the experiment, but for the smaller mean particle sizes this same trend fades out. The X-ray absorption spectra of these samples have nominally the same signal to noise ratio (see for example Supplementary Fig. 17). Secondly, we see that there is always a degree of restructuring that seems to be irreversible (in the beginning the change is always largest). The third observation is that larger particles have a more significant slope for ethene-induced restructuring, and hence restructure faster, than smaller particles do. As the variation of Δ_t_σ^2^ is derived from the EXAFS shown e.g., in Figs. 1 and 2, the integral of the rate of restructuring, can be related to the overall magnitude of restructuring to ensure we did not miss, for example, very fast restructuring in the small nanoparticles which does not seem to be the case. Our observation that larger particles restructure faster than small ones is unexpected, as the more open, or stepped surfaces (i.e., smaller nanoparticles) are thought to be the less rigid^36^. Indirect observations in literature have also pointed towards smaller particles spontaneously restructuring faster^37^. It is interesting to note, however, that carbon diffusion in Ni was reported to be highly anisotropic due to the difference in energy barrier for migration parallel to grain boundary (*E*_a_ = 42–74 kJ mol^−1^) versus lattice diffusion (*E*_a_ = 160 kJ mol^−1^)^38^. Such a difference in activation energy at 150 °C results in approximately 10^10^ times faster kinetics, such that C migration along grain boundaries may happen in the order of the seconds that we record. We therefore argue that the larger Δ_t_σ^2^ change observed for the larger nanoparticles could be explained by C diffusion in the bigger nanoparticles along grain boundaries, which were observed by HRTEM (see Supplementary Fig. 31).

### The speed of carbon intercalation in nanoparticles

**Fig. 4:**
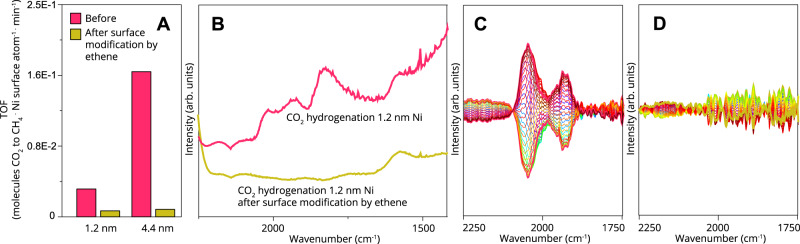
Surface expansion onset by ethene flow leads to structure insensitivity for structure sensitive reaction. **a** Turnover frequency of CO_2_ hydrogenation over two Ni catalyst samples with differing particle size, before and after surface modification with ethene. **b** FT-IR spectra of Ni sample with 1.2 nm mean Ni particle size in CO_2_ hydrogenation before and after surface modification with ethene. **c**, **d** DRIFTS phase domain spectra of CO_2_ modulation excitation experiment **c** before and **d** after surface modification with ethene.

In fact, the time resolved distortion of the nanoparticle structure induced by ethene hydrogenation, can yield insight into the intercalation of C species into our metal nanoparticles, which seems to occur particle size-dependently for this structure insensitive reaction. The perturbation of our nanoparticles by C can be qualitatively compared (by the dimensionality arguments) to the diffusion coefficient of carbon in Ni. This, in turn, can be evaluated via the Einstein diffusion equation (see Supplementary Note 4). In fact, the derivative of the mean-square relative displacement (MSRD) σ^2^ over time (Fig. 4a, b, and Supplementary Figs. 18–20), can be related, by dimensionality, to the diffusion coefficient of C in Ni. A deeper look into this process helps extend the connection between the species and processes involved in the carbon-induced perturbation of our nanoparticles. Evidently, two processes take place in Fig. 3b. One sharp peak, a fast, physical process (i.e., relatively low E_a_ barrier) with the onset of an ethene pulse, and one less sharp slope (i.e., relatively high *E*_a_ barrier), during the same ethene pulse. By taking the maximum of the first peak for each nanoparticle size in Fig. 3b and comparing the found values to literature, we find firstly that the carbon diffusion coefficients are in the same order of magnitude as one would expect based on the equation of Lander et al. (with an average of 3.5 × 10^−21^)^39^, and secondly that the calculated carbon diffusion coefficient increases with mean Ni particle size. That is, the larger the particle, the faster C apparently diffuses for ethene hydrogenation experiments. Considering the match in C diffusion from the experiments and literature, and knowing that the respective activation energy barriers for C diffusion are in the range of 120–150 kJ/mol^39^ (as with the diffusivity, activation energy barriers can be calculated), we rationalize that the other slower physical process must have a larger E_a_ as activation energy barriers are directly related to rate of occurrence. Such a slower process, for example, could be the deformation of particle shape or the removal or positional distortion of atoms from a surface facet. For Ni, these occurrences could be on the order of 150–428 kJ/mol, calculated by taking the cohesive energy of Ni divided by 12 (bulk), multiplied by a number of different adatom formation possibilities^15^. Hence, we can say that the surfaces of our catalytic nanoparticles are likely distorted by examining the perturbations observed by XAS.

To prove that this is the case, one may deduce the volume fraction of C that is intercalated into the metal nanoparticles, from the time resolved quick-XAS experiments, and different particle sizes, and the respective disorder factors. The calculated diffusion values indicate that, in a time in the order of 10 s (in which we observe the variation of ${\sigma }^{2}$), the nuclear mean square displacement of C is in the order of 1 × 10 Å^−2^, and since this is much smaller than a Ni-Ni distance (2.5 Å) carbon should only occupy surface sites in our case. Nonetheless, as Supplementary Fig. 26 shows, the 1.2 nm NPs have the smallest change in the ${\sigma }^{2}$ values, and the 4.4 nm the greatest, with a ratio of

1 $${\triangle \sigma }^{2}(1.2)/{\triangle \sigma }^{2}(4.4)=0.76$$

This is unexpected since the ratio of the surface sites in 1.2 nm and 4.4 nm nanoparticles is

2 $${x}_{{surf}}(1.2)/{x}_{{surf}}(4.4)\approx 0.5/0.15=3.3$$

indicating that either the surface of the smaller nanoparticles is not entirely restructured or that the bigger nanoparticles are restructuring also (partially) in their bulk. See also Supplementary Note 5 for more details on this calculation. An excellent way to check if these are carbidic-type (defined here as C adatoms – not necessarily an ordered periodic carbide structure) or C_2_ reaction intermediates that are restructuring the surface, is to check the formation of odd-C_x_-numbered oligomers in our GC data. If we examine the product distribution of our Ni catalysts with different particle sizes in ethene hydrogenation, we can see that every catalyst (see Fig. 3c–e) converts a small amount of ethene to methane, implying that there is at any given point in time also molecular carbon (i.e., carbidic), or CH_x_ fragments, on the surface.

The more significant restructuring for larger particles thus likely reflects more bulk carbide formation. The fact that we see product formation at similar turnover frequencies for the operando quick-XAS experiments (see Fig. 3c–e, and Supplementary Fig. 22) as reported in literature^23^ and our experiments in Fig. 1, indicates that our catalyst is not simply poisoned by these carbon fractions. It is important to note that as such, these C-induced perturbations (whether they be on the surface or in the bulk of the nanoparticle), do not poison catalyst activity.

### In-situ STEM corroborates at the single-particle level

The results that have been discussed up until now all consider bulk measurements (FT-IR, quick-XAS). To assess whether similar results could be found at the single nanoparticle level, in-situ high resolution scanning transmission electron microscopy (HR-STEM) measurements were performed. Section ‘Supplementary Methods’ of the Supporting information lists details on sample preparation and experimental procedures. Figure 3f–h (and Supplementary Figs. 29–33) shows in-situ HR-STEM results of Ni nanoparticles directly deposited onto SiN membranes via spark ablation of Ni electrodes (see ‘Supplementary Methods’ section on spark ablation). The average lattice spacing of the Ni nanoparticles was measured directly on several nanoparticles using Fast Fourier Transform (FFT) in H_2_, subsequently in flow of ethene, and once more in H_2_ (see Supplementary Figs. 29–33)_._ Upon the introduction of ethene, an average surface expansion of +1.7 ± 0.9% was measured (over ~4.5 nm^2^ in 5 separate particles each existing of at least approximately 8.000 Ni atoms based on the nanoparticle size in nm^14^) while the subsequent contraction upon the introduction of hydrogen was −1.6 ± 0.9%. Therefore, indeed, also some intercalation of carbon must occur under ethene environment as STEM is not a surface sensitive technique.

In light of the fact that both the bulk and single particle levels showed lattice expansion upon the introduction of ethene hydrogenation, it is now interesting to discuss whether we can relate these observed dynamic surface perturbations to structure insensitivity. Regardless of the interesting question about what exact type of strongly bound C is restructuring the Ni surface so significantly, it is clear that the impact of these species is different for different catalyst particle sizes (e.g. Figs. 1h and 3b). Somorjai and coworkers determined that an ethylidyne intermediate (C_2_H_3_) is very strongly bound to the surface via a threefold carbon metal bond under specific reaction conditions^40^. This specific strong adsorption can create trenches in which a row of surface atoms is exposed over which π-bonded ethene (the proposed active intermediate in ethene hydrogenation) reacts^8,41,42^. Imagining such a type of carbon species adsorbing on the surface could thus mean that a predetermined fraction of sites may be exposed no matter what the previously exposed surface consists of. For ethene hydrogenation, upon the ethylidyne-induced creation of these trenches, a surface expansion of approximately 1.5%–2% is expected^36^.

### Apparent structure insensitivity

The experiments we show here clearly imply that the structure insensitive heterogeneous catalytic reaction ethene hydrogenation restructures different mean Ni nanoparticle sizes significantly but also *particle size dependently*. If there is still surface exposed after this ethene restructuring, and these exposed sites are of a specific site composition, then this can cause the observed structure insensitivity. To check these parameters, we performed a probe reaction with the goal to examine the effect of restructuring induced by ethene hydrogenation on a subsequent catalytic reaction, CO_2_ hydrogenation. Figure 4a shows the surface-normalized activity of 2 different Ni nanoparticle sizes in CO_2_ hydrogenation. Before surface modification by ethene, as plotted in Fig. 4a in pink, the two different particle sizes show significantly distinct particle size dependent activity, previously shown to be caused by the availability of different sites with different activity on these same catalytic samples^29^. The same catalyst samples were then exposed to ethene hydrogenation and subsequent hydrogenation of the catalyst surface, after which only irreversibly adsorbed C should be adsorbed on the surface (Fig. 3b). After this, CO_2_ hydrogenation was performed once more on the catalyst nanoparticles. The surface-normalized activity decreased significantly after this ethene-perturbation and was nearly indistinguishable for the two types of nanoparticles as can be seen in Fig. 4a. In other words, our previously structure sensitive reaction had become structure insensitive, while still being catalytically active.

Figure 4b–d show FT-IR spectroscopy experiments on the effect of this surface modification with ethene. Figure 4b–d demonstrate that CO_2_ activation does still occur, and that CO_ads_ species are formed and consumed on the surface upon pulses of CO_2_. If there is still activity, and we still see CO-Ni vibrations (as we do), some sites are apparently left free by surface modification. This suggests that the restructuring onset by ethene is a structure sensitive occurrence—and the observed changes in activity are not just a poisoning effect. The ethene-onset restructuring exposes a certain fraction of sites which, apparently, make CO_2_ hydrogenation structure insensitive (Fig. 4a).

In light of these considerations, it is interesting to note that the most likely explanation for empirically observed structure insensitivity *is not* the absence of sensitivity to geometrical and electronic effects as generally assumed. We do wish to point out that even if ethylidyne, or any other surface carbon is the actual active intermediate in ethene hydrogenation, this reaction is “structure insensitive” for the trivial reason that the reaction does not proceed on the exposed metal sites and also in this case, structure insensitivity (that is, the absence of electronic and geometrical effects on a catalytic reaction) as often currently described in literature does indeed not exist. Rather, if structure insensitivity is empirically observed, it is caused by a surface that cannot simply be considered as the superposition of contributions from individual crystal planes.

As far as we are aware, this is the first report of the direct observation of particle size-dependent restructuring which is shown to increase with particle size, and of faster restructuring of larger metal nanoparticles. The interesting notion that there is always a degree of restructuring which is irreversible, and the proofs provided for more and faster intercalation of carbon for larger nanoparticles have obvious implications for the catalytic reactions that follow on such metal nanoparticles particles. Although our findings are based on experiments with Ni nanoparticles supported on SiO_2_ and two specific catalytic reactions, we believe effects of similar nature are to be found (to larger or smaller extents) throughout reactions on supported metal particles and thus we believe these experiments also to be relevant to interface chemistry and related fields.

## Methods

### Catalyst synthesis

Silica supported Ni nanoparticles were made by homogeneous deposition precipitation (HDP), and coprecipitation according to e.g. Ermakova et al.^43^ or de Jong and Lok^44,45^. All catalysts (see Supplementary Information) were prepared by HDP except 6.0 nm Ni/SiO_2_. To this end different weight loadings (1–20%) of Ni precursor were brought in solutions along with suspended silica, and pH was increased under continuous stirring by addition of an alkaline solution. Sample 6.0 nm Ni/SiO_2_ was made by coprecipitation of NiCO_3_ with silica precursor in solution, precipitation was onset by addition of NaOH. The catalyst samples under investigation have varying Ni mean particle sizes, as listed in Supplementary Table 3.

### Catalyst characterization

All details on catalyst characterization can be found elsewhere^29^.

### Operando FT-IR with on-line product analysis

Operando Fourier transform infrared (FT-IR) spectroscopy measurements were performed to study a structure sensitive reaction (CO_2_ hydrogenation) and a structure insensitive reaction (ethene hydrogenation) over SiO_2_ supported Ni catalysts with varying particle size. Product formation was followed by on-line gas chromatography. Before each reaction, catalysts were reduced (see also section ‘Supplementary Methods’ in the Supplementary Information. The overall procedure and setup has been described elsewhere^29^.

### Operando quick-XAS with on-line product analysis

Pulsed operando X-ray absorption spectroscopy experiments were performed at the SuperXAS beamline (X10DA) at the Swiss Light Source in transmission mode. The X-ray beam from the 2.9 T bending magnet was collimated by a Si coated mirror and monochromatized with a Si(111) channel-cut crystal in the QuickXAS monochromator. The Si(111) crystal was rotated at a frequency of 10 Hz across the Ni K-edge, and the signals of the ionization chambers and the angular encoder were sampled at a frequency of 2 MHz. The edge energy for the Ni spectra was calibrated using a Ni foil. The measurements were performed in a custom-built operando reaction cell^29^. Q-XAS data was subsequently evaluated using the JAQ Analyzes QEXAFS version 3.3.53 software as well as self-developed Matlab™ code, preparing the data for batch fitting with Larch FEFFIT. Further information about the Q-XAS data processing can be found in the Supplementary information.

### In-situ STEM

The in-situ STEM experiments were performed at the ORNL Center for Nanophase Materials Sciences using a Protochips Atmosphere in-situ gas cell system and a probe corrected FEI Titan S/TEM operating at 300 kV. Samples were prepared using the spark ablation technology of VSParticle, by use of their G1 nanoparticle generator (see section ‘Supplementary Methods’ of the Supporting information). The nanoparticles were directly deposited onto the SiN membrane of the Protochips MEMS reactors using the following parameters; 99.99% Ni, (Chempur), Argon 5.0 (Linde), voltage 1.3 kV, current 8.1 mA, flowrate 10 SLM, deposition time 6 min. The nanoparticles were heated under H_2_ and ethene gas to 150 °C at atmospheric pressure and imaged using bright-field (BF) and annular dark-field (ADF) STEM imaging.

## Supplementary information

Supplementary Information

## Data Availability

The datasets generated during and/or analyzed during the current study are available from the corresponding author on reasonable request.

## Code availability

The code generated to analyze the datasets during the current study are referenced, or available from the corresponding author on reasonable request.
