# Supplementary material for: Dynamic restructuring of supported metal nanoparticles and its implications for structure insensitive catalysis
Source: Nat Commun. 2021 Dec 7;12:7096. doi: 10.1038/s41467-021-27474-3 (PMC8651646; doi:10.1038/s41467-021-27474-3)
Supplement: Supplementary file 1 — Supplementary Information [file 41467_2021_27474_MOESM1_ESM.docx]

Supplementary Information to: Dynamic Restructuring of Supported Metal Nanoparticles and its Implications for Structure Insensitive Catalysis

Vogt et al.

CONTENTS

[Supplementary Note 1 – Structure Sensitivity versus Structure Insensitivity 3](#_Toc87372368)

[Supplementary Methods 8](#_Toc87372369)

[Supplementary Note 2 – Ethylene Hydrogenation Catalysis 22](#_Toc87372379)

[Supplementary Note 3 - CO_2_ Hydrogenation Catalysis 24](#_Toc87372380)

[Supplementary Discussion - X-ray Absorption Spectroscopy 25](#_Toc87372381)

[Supplementary Discussion - In-situ HR-TEM 39](#_Toc87372384)

[Supplementary Note 4 – Estimate of the Diffusion Coefficient of C through Ni Using the Time-Dependent Reaction Data 42](#_Toc87372385)

[Supplementary Note 5 – Estimate of the Volume Fraction of C Intercalation Sites in Ni Nanoparticles from their Disorder Parameters Measured by EXAFS 45](#_Toc87372386)

[Supplementary References 47](#_Toc87372387)

# Supplementary Note 1 – Structure Sensitivity versus Structure Insensitivity

One of the classifications in catalysis that seems to have withstood decades of research is one that divides catalytic reactions into one of two groups; reactions that are empirically found to be either structure sensitive, or structure insensitive. In the former, the catalytic turnover per unit surface area (e.g. the turnover frequency, TOF) changes with the metal particle size of the catalyst. Conversely, reactions in which no such change in turnover frequency with metal particle size or crystal facet can empirically be observed are termed structure insensitive.

Supplementary Table 1 lists the available literature that mentions structure sensitive or insensitive reactions over nickel catalysts. It can be seen in this Table that the classification of structure sensitivity has also been made based on differing turnover rates per single crystal facets. Two reactions that were found to be structure insensitive for nickel catalysis (CO methanation and ethylene hydrogenation^1^) were tested on two terrace Ni facets, Ni(111) and Ni(100). Using information we have today, these classifications should not have been made without also including a stepped facet in the study, and preferably by studying different particle sizes. The other two reactions that were found to be structure insensitive (the hydrogenation of benzene and of 2-ethyl-hexen-2-al^2,3^) were tested on supported Ni catalysts with different weight loading without verifying Ni particle size. Very few reports are made on structure insensitive reactions versus structure sensitive ones. As can be seen in Supplementary Table 1, quite some were later reclassified to be structure sensitive after originally being classified as structure insensitive.

Structure sensitivity, as opposed to structure insensitivity, is arguably quite well-understood. That is, it is often explained as σ-bonds are preferentially cleaved over highly undercoordinated sites, while π-bonds need a certain (optimal) degree of site coordination leading to varying turnover numbers on varying particle sizes which have different ratios of these facets exposed. Convoluted mixtures of these two explanations are often the reality in real catalytic reactions consisting of many sequential reaction steps, along with for example support-interface effects, or steric considerations. Generally, structure sensitivity can be explained by electronic and geometric effects which are correlated but can nevertheless be separated based on Brønsted-Evans-Polanyi relationships^4^.

These generalizations don’t just hold for nickel, however. Supplementary Table 2 lists an overview of well-established studies on catalytic reactions on different metals, their classification of structure (in)sensitivity, and it also lists a brief explanation of the observed structure-activity relationship. Again, looking through Supplementary Table 2, it can be seen that for quite some reactions which are termed structure insensitive, studies claiming structure sensitivity for the same reaction also exist.

In fact, often the classification of structure insensitivity is made based on the reaction being “much less sensitive to structure” than a given counterpart^21^. It has even been noted that no true examples of structure insensitivity can be found^4^. Structure sensitivity and insensitivity trends (i.e. that which is related to geometric and a specific type of electronic effect^4^) occur in the region where no quantum effects occur^22^. While some interesting recent studies touch upon the interesting effects of sub-nanometer size nanoparticles^23,24^, the quantum effects that come to play here are outside of the scope of the effect that is described in this study.

By examining the explanations from the different studies for empirically noted structure insensitivity from Supplementary Table 2 we can observe an interesting trend. For each reaction, the leading explanation to structure insensitivity is an erasure of previously existing surface site anisotropy either by surface reconstruction^25^, a carbonaceous overlayer^26^, or e.g. CO saturation of the surface^27^.

In conclusion, the true structure insensitivity of catalytic reactions has never been proven, and there is significantly more evidence against it than for it. Structure sensitivity, conversely, is well-established.

Nevertheless, empirically, structure insensitivity can still be observed. Thus to study these phenomena, in an attempt to understand structure insensitivity better, we proceeded to take a classic structure sensitive (CO_2_ hydrogenation) and classical “structure insensitive” reaction (ethene hydrogenation) and study it over a number of supported Ni catalysts with varying mean particle size.

The classification of CO_2_ hydrogenation as a structure sensitive reaction can be found throughout the open literature, as can be seen in Supplementary Figure 1 which also shows one of the most widely studied structure sensitive reactions - Fischer-Tropsch synthesis - as a comparison. Supplementary Figure 1 also shows ethene hydrogenation (1:1 ethene:H_2_) which is classically accepted to be a structure insensitive reaction^23^. Via this literature search for non-single crystal facet studies of ethene hydrogenation, we can see that even for this classical example of a “structure insensitive” reaction; structure sensitivity can still be observed in some studies. It should be clear from all of the above that structure insensitivity in this most classical example may not be as well established as we often assume in literature. Furthermore, it should be noted that zero TOF dependence on particle size may be something that is approached only under certain conditions.

As mentioned in the main text, in order to establish the structure sensitivity trends of these reactions, a set of silica-supported Ni nanoparticular catalysts was synthesized. Supplementary Figure 2 shows the influence of Ni mean particle size on the surface-normalized activity for CO_2_ hydrogenation (1:4 CO_2_:H_2_) which is the structure sensitive reaction under study. Both data from a previous publication^9^, and data collected to supplement previous data for this work is shown both separately, and together, plotted on a logarithmic, and linear Y-axis. The TOFs were calculated based on particle size determination by HAADF-STEM, H_2_ chemisorption, and X-ray absorption spectroscopy^9^.

Supplementary Figure 3 shows the structure sensitive and insensitive reactions (as shown in Figure 1 of the main text) plotted on a logarithmic Y-axis.

Supplementary **Table 1.** Overview of structure (in)sensitive reactions in literature using nickel catalysts.

| **Reaction** | **Catalyst** | **Structure (In)Sensitivity** | | **Ref** |
| --- | --- | --- | --- | --- |
| Benzene hydrogenation | SiO_2_ supported Ni-Cu NPs with varying particle size | | Structure sensitive | ^5^ |
| CO methanation | Single crystal Ni(111), supported Ni NPs | | Structure sensitive | ^6^ |
| CO methanation | Supported Ni NPs | | Structure sensitive | ^7^ |
| CO methanation | Single crystal Ni(111), Ni(100) and 1 sample of Al_2_O_3_ supported Ni NPs | | Structure insensitive | ^1^ |
| CO methanation | SiO_2_ supported Ni NPs with varying particle size | | Structure sensitive | ^8^ |
| CO_2_ methanation | SiO_2_ supported Ni NPs with varying particle size | | Structure sensitive | ^9^ |
| CO_2_ methanation | SiO_2_, Al_2_O_3_, TiO_2_, CeO_2_, ZrO_2_ supported Ni NPs with varying particle size | | Structure sensitive | ^10^ |
| Cyclopropane hydrogenation | Single crystal Ni(111), Ni(100) and Al_2_O_3_ supported Ni NPs | | Structure sensitive | ^1^ |
| Dehydrogenation and dehydration of formic acid | SiO_2_ supported Ni NPs with varying particle size | | Structure sensitive | ^11^ |
| Dry methane reforming | SiO_2_ supported Ni NPs with varying particle size | | Structure sensitive | ^12^ |
| Ethane hydrogenolysis | Single crystal Ni(111) and Ni(100) | | Structure sensitive | ^13^ |
| Ethane hydrogenolysis | Single crystal Ni(111), Ni(100) and Al_2_O_3_ supported Ni NPs | | Structure sensitive | ^1^ |
| Ethylene hydrogenation | SiO_2_ supported Ni NPs with varying particle size | | Structure sensitive | ^14^ |
| Ethylene hydrogenation to methane | Single crystal Ni(111), Ni(100) and 1 sample of Al_2_O_3_ supported Ni NPs | | Structure insensitive | ^1^ |
| Hydrodechlorination of chlorobenzene | SiO_2_, Al_2_O_3_, MgO supported Ni NPs with varying particle size | | Structure sensitive | ^15^ |
| Hydrodesulfurization of 4,6-dimethyldibenzothiophene | SiO_2_ supported Ni NPs with varying particle size | | Structure sensitive | ^16^ |
| Hydrogenation of benzene | Al_2_O_3_ supported Ni with varying weight loading | | Structure insensitive | ^2^ |
| Hydrogenation of methylacetoacetate | Ni-exchanged Y zeolite catalysts with varying Ni particle sizes | | Structure sensitive | ^17^ |
| Hydrogenation of 2-ethyl-hexen-2-al | Al_2_O_3_ supported Ni with varying weight loading | | Structure insensitive | ^3^ |
| Hydrogenolysis of n-butane | SiO_2_ supported Ni NPs with varying particle size | | Structure sensitive | ^14^ |
| Methanol decomposition | SiO_2_ supported Ni NPs with varying particle size | | Structure sensitive | ^18^ |
| NH_3_ decomposition | Al_2_O_3_ supported Ni NPs with varying particle size | | Structure sensitive | ^19^ |
| Propane hydrogenolysis | Single crystal Ni(111), and SiO_2_ supported Ni NPs | | Structure sensitive | ^20^ |
| Steam methane reforming | SiO_2_ supported Ni NPs with varying particle size | | Structure sensitive | ^12^ |

**Supplementary Table 2.** Overview of structure (in)sensitive reactions in literature using catalysts of different metals.

| **Catalytic reaction** | **Classification** | **Catalyst** | **Explanation** | **Reference** |
| --- | --- | --- | --- | --- |
| Alkyl hydrogenation | Structure insensitive | Pt(111) | Structure insensitive because of a surface carbon overlayer, or the surface that was exposed has restructured in catalytic equilibrium erasing any previous surface anisotropy. | ^28^ |
| Aromatic hydrogenation | Structure insensitive | Al_2_O_3_, TiO_2_ supported Ru, Pt and Rh nanoparticles | No explanation is given, but a carbon overlayer caused by the large aromatic molecules could explain structure insensitivity. Furthermore, it should be noted that a degree of structure sensitivity was indeed found for benzene hydrogenation, while naphthalene hydrogenation was less sensitive to particle size in this study. | ^21^ |
| Fischer Tropsch synthesis (CO hydrogenation to C_2+_ hydrocarbons) | Structure sensitive | CNT supported Co nanoparticles | CO is preferentially cleaved on B_5_ sites. | ^29^ |
| CO hydrogenation (to C_1_ hydrocarbons) | Structure insensitive | Rh, Pd, Ni(111), Rh, Pd, Ni (100) | CO saturation of the surface, the few remaining sites can erase surface anisotropy altogether. | ^30^ |
| CO_2_ hydrogenation (Sabatier reaction) | Structure sensitive | Carbon supported Pt  SiO_2_ supported Ni | There should be an optimal interplay between the sites that hydrogenate (σ-bond) C (and CO), and the cleavage of the first O from CO_2_ (π-bond). | ^31^  ^9^ |
| Steam methane reforming | Structure sensitive | SiO_2_ supported Ni  Al_2_O_3_, ZrO_2_ supported Rh | CH_4_ cleavage is structure sensitive (highly uncoordinated sites, σ-bond), but for very small nanoparticles CO formation (π-bond) can become limiting | ^12^  ^32,33^ |
| Ammonia synthesis | Structure sensitive | Fe(111), Fe(100), Fe(110) | The activation of N_2_ (π-bond) is extremely structure sensitive | ^34^ |
| Ethane hydrogenolysis | Structure sensitive | Pt(111) | Reverse structure sensitivity as C-C bond cleavage is dependent on CH activation (which is structure sensitive) | ^28^ |
| Carbonylation | Structure sensitive | TiO_2_-SiO_2_ supported Cu | Recombination of adsorbed surface species is the rate limiting step. | ^35^ |
| Hydroformylation | Structure sensitive | SiO_2_ supported Rh | For the carbonyl-derived supported Rh catalysts, undercoordinated sites showed much higher hydroformylation rates, likely largely due to a geometric effect. | ^36^ |
| CO oxidation | Structure insensitive  Structure sensitive | Pd(111), Pd wire, Pd/Al_2_O_3_ crystal  Surface science: Rh(111) vs Rh(100) | CO saturation of the surface, the few remaining sites might have erased surface anisotropy altogether.  CO* and O* destabilization on Rh(100) versus Rh(111) (with increasing coverage) | ^27^  ^37^ |


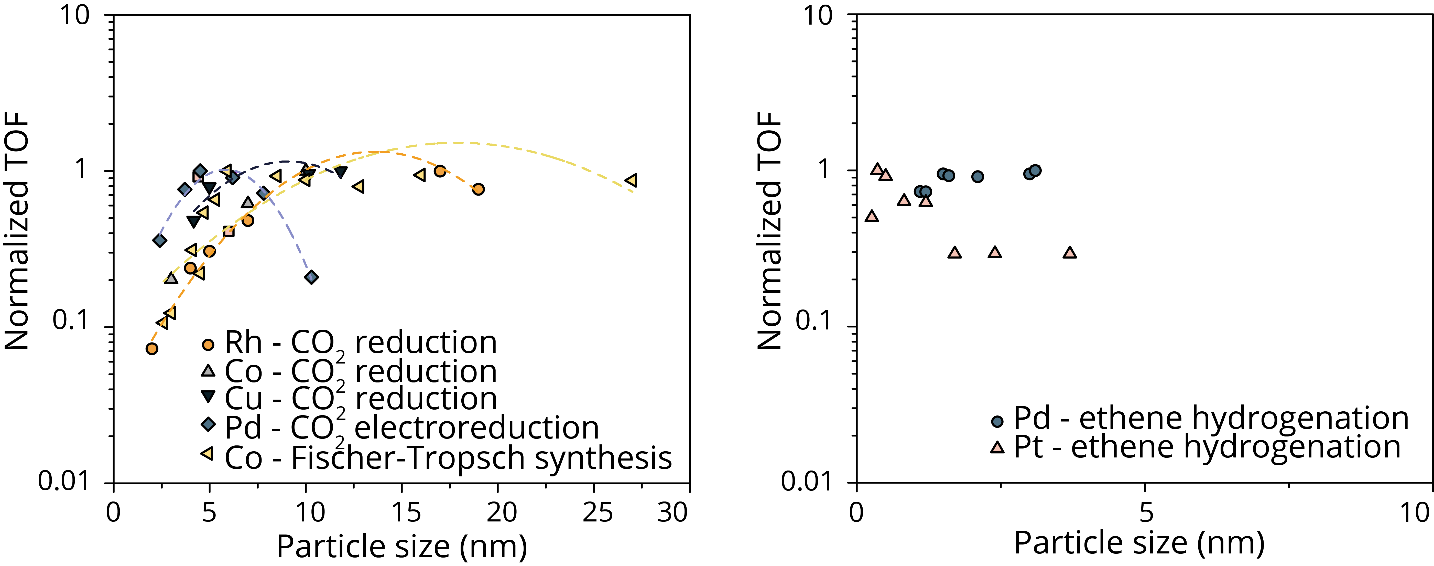


Supplementary Figure 1. Literature data available on CO_2_ hydrogenation, and related π-type structure sensitive reaction Fischer-Tropsch synthesis, and on classical structure insensitive reaction ethene hydrogenation. The quadratic eye-guides in the left panel are drawn purely to show which data belongs to the same study.

# Supplementary Methods


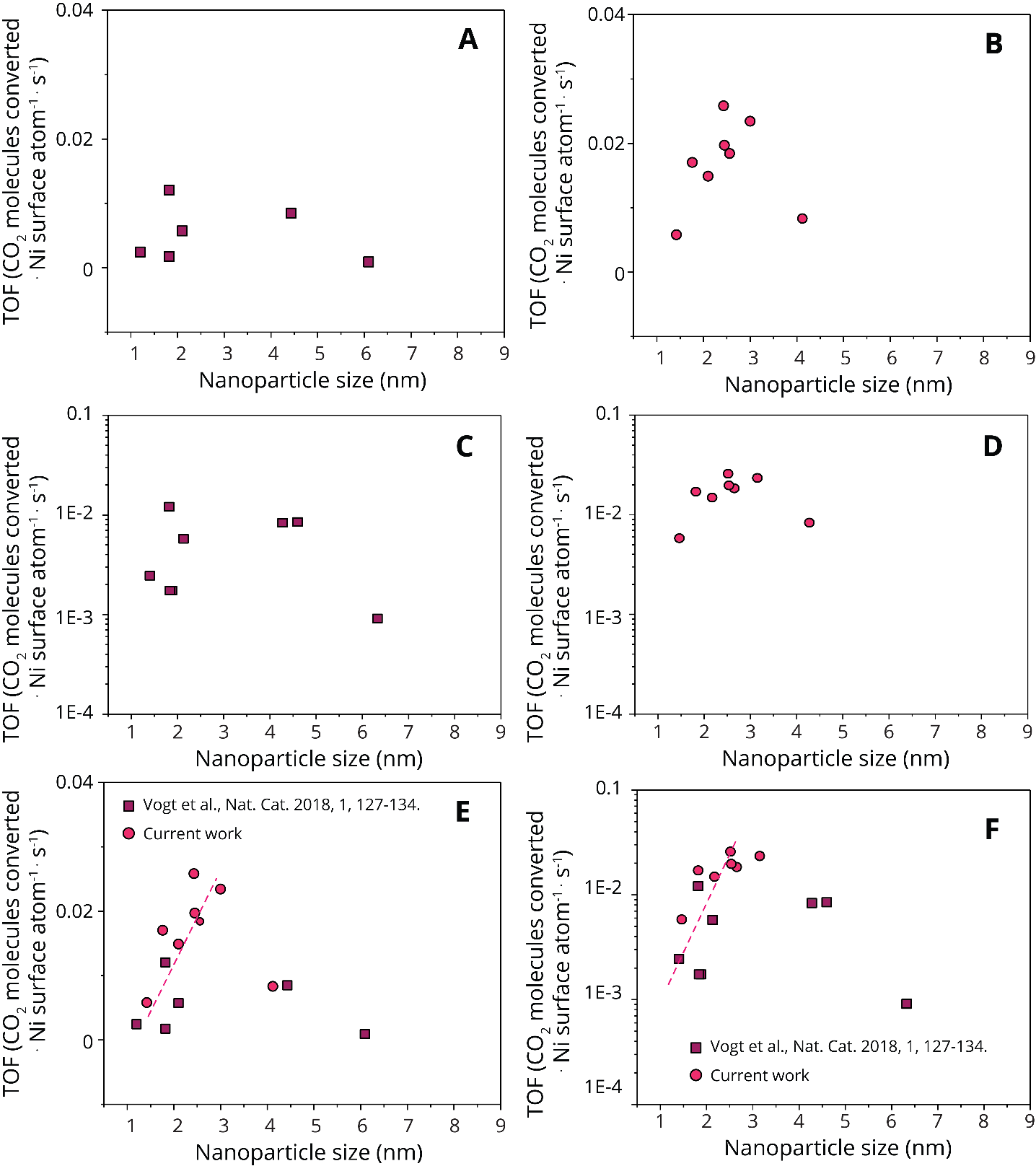


Supplementary Figure 2. The influence of Ni mean particle size on the turnover frequency (TOF). A, C, show data from a previous publication on a linear (A) and logarithmic axis. B, D, show new data collected to supplement existing data for this work on a linear (B) and logarithmic scale (D). E, F, show the data combined on a linear (E) and logarithimic (F) scale.

Supplementary Figure 3. Structure sensitivity versus structure insensitivity plotted on a logarithmic Y-axis. The influence of Ni mean particle size on the TOF for (A) CO_2_ hydrogenation at 300 °C and (B) ethene hydrogenation at 150 °C.


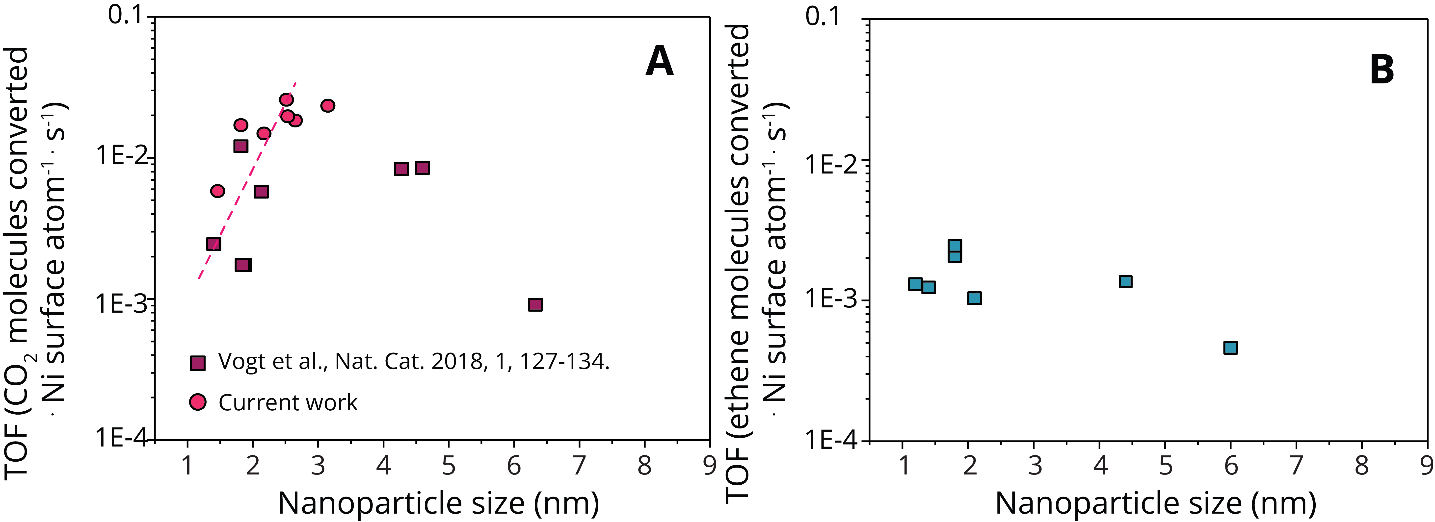


Supplementary Table 3. Catalyst samples used in this study. Main samples under study (*). Full characterization details can be found elsewhere^9^.

| **Ni mean particle size on SiO_2_ from HAADF-STEM after reduction^1^ (used as catalyst code)** | **Standard deviation in column 1 (nm)** | **Ni weight loading (%)** |
| --- | --- | --- |
| 1.2 nm * | ± 0.5 | 4.7 |
| 1.4 nm | ± 0.4 | 5 |
| 1.8 nm | ± 0.3 | 1.8 |
| 1.8 nm | ± 0.8 | 6.7 |
| 2.1 nm * | ± 1.1 | 11.8 |
| 2.4 nm | ± 0.6 | 5 |
| 2.5 nm | ± 0.8 | 1.6 |
| 2.6 nm | ± 0.9 | 1.6 |
| 3.0 nm | ± 0.6 | 1.6 |
| 4.1 nm | ± 1.0 | 30 |
| 4.4 nm * | ± 2.4 | 19.5 |
| 6 nm | ± 1.9 | 60 |

Silica-supported Ni nanoparticular catalysts were prepared by varying the concentration of the Ni precursor solution in deposition precipitation or coprecipitation (see Supplementary Table 3). Catalysts with mean particle sizes of 1-5 were prepared via deposition precipitation. The catalyst sample with Ni mean particle size of 6 nm was prepared via coprecipitation. Details on catalyst characterization can be found elsewhere in literature, and below for samples marked with an asterisk*^9^.

### Characterization results

The following extensive characterization results on the SiO_2_-supported Ni samples with mean particle sizes 1.2, 2.1, and 4.4 nm can also be found in Vogt et al. ^9^, and are reported here merely for the readers’ convenience.

### Ex-situ x-ray absorption spectroscopy

The nickel oxidation state of each of the catalyst samples under investigation (A-H) was investigated by X-ray absorption spectroscopy (XAS). Fingerprinting the Fourier transform magnitude of the phase-corrected EXAFS (R space) showed that the samples, using the reduction method as described previously, were >90% reduced, with a 10% experimental error.

Supplementary Figure 4. Fourier-transform magnitude of the phase-corrected EXAFS data of a Ni-foil reference, and the various nickel catalyst samples after the reduction procedures described.


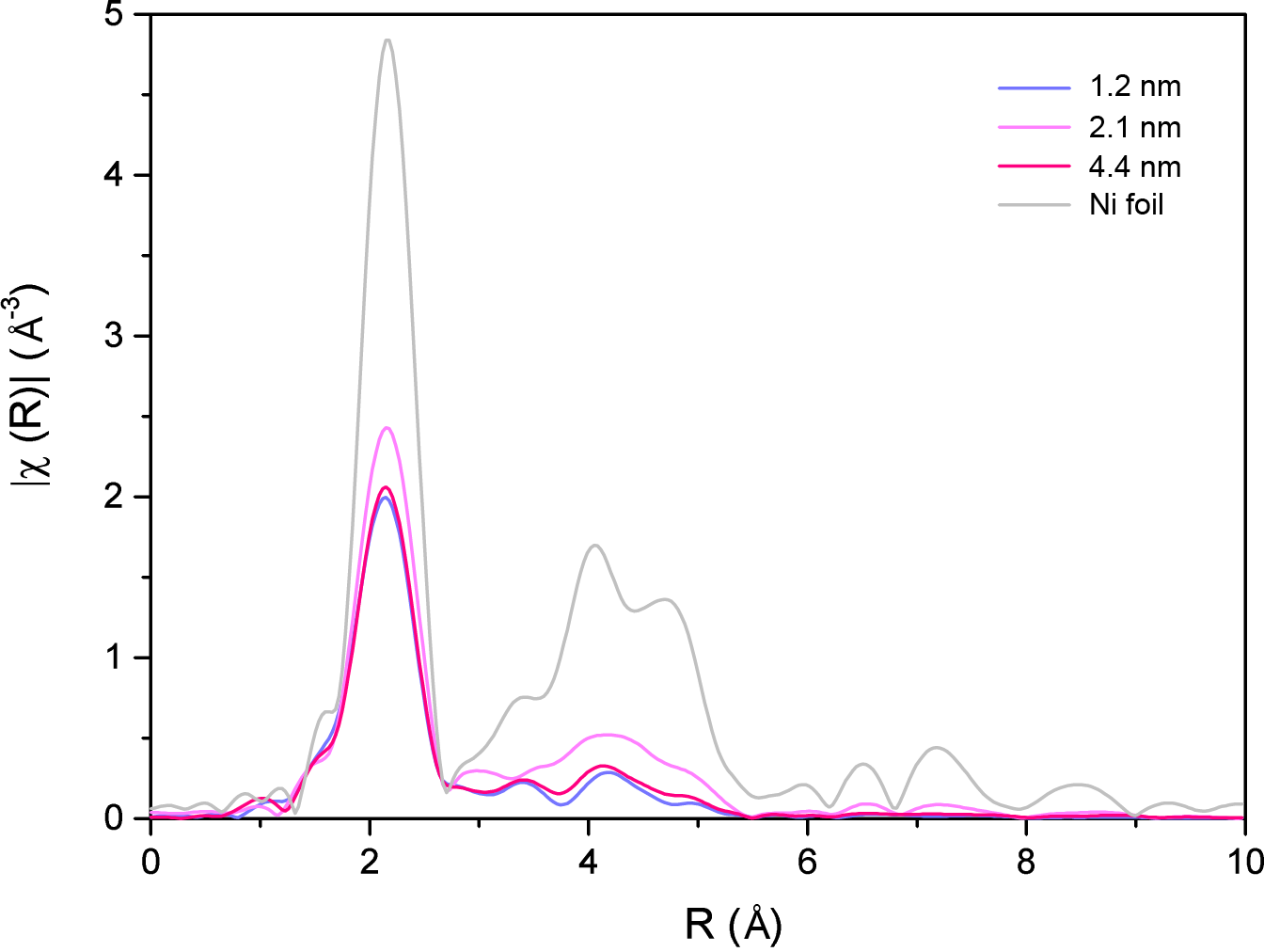


####

### HAADF-STEM of post-reduction (Ni) particle sizes

Materials for examination by scanning transmission electron microscopy (STEM) were dry dispersed onto a holey carbon TEM grid. The catalyst samples were examined using bright field (BF) and high angle annular dark field (HAADF) imaging modes in an aberration corrected JEOL ARM-200CF scanning transmission electron microscope (STEM) operating at 200 kV. This microscope was also equipped with a Centurio silicon drift detector (SDD) system for X-ray energy dispersive spectroscopy (XEDS) analysis.

The Ni/SiO_2_ catalysts subjected to STEM analysis had undergone a reduction step at the beginning of the catalytic reaction. However, the Ni was subsequently re-oxidised upon exposure to air under ambient conditions during storage and transfer into the electron microscope. It was found that smaller particles (< ~3 nm in diameter) were fully oxidized to cubic NiO as shown in Supplementary Figure 5A-B. In contrast, larger particles (> 3 nm in diameter) possessed a metallic Ni core and an oxidized shell morphology (see Supplementary Figure 5C-D). The NiO shells of the larger particles exhibited relatively uniform thickness, with a mean thickness of 1.36 ± 0.24 nm.

Hence when measuring particle size distributions by *ex-situ* methods such as TEM, STEM or XRD, we were always sampling the Ni particle after it had been fully or partially oxidised to NiO. However, we were able to correct for this effect in our STEM data, by back calculating the size of the original Ni particle that would be needed to generate a NiO or Ni/NiO core-shell particle of a certain dimension. Hence from the measured particle size distributions we were able to produce more meaningful histograms of the original Ni particle size, which would be more relevant to the catalyst under actual working conditions. These corrected Ni particle sizes were used for calculating activity and TOF values of the various catalysts.

Experimentally, the equivalent spherical diameter was taken as the particle diameter, which was deduced by measuring the 2-D projected area of the particles from HAADF-STEM images. We also took the average shell thickness, *t*, to be 1.36 nm. If we denote the measured diameter of the whole particle to be d_measured_, and assume the lattice constants of metallic Ni and NiO to be a_Ni_=0.353 nm and a_NiO_=0.418 nm respectively, we can then estimate the diameter for the original metallic Ni particle as:

Eq. 1 $\left\{ \begin{matrix} Fully oxidized particle \left( d_{\mathrm{whole}}\leq2t \right): & d_{\mathrm{original}}=d_{\mathrm{measured}}\times\left( {a_{\mathrm{Ni}}}/{a_{\mathrm{NiO}}} \right) \\ Partially oxidized particle \left( d_{\mathrm{whole}}>2t \right): & d_{\mathrm{original}}=\left( d_{\mathrm{measured}}-2t \right)+2t\times\left( {a_{\mathrm{Ni}}}/{a_{\mathrm{NiO}}} \right) \end{matrix} \right.$

The number of particles analyzed by STEM for each sample is listed in Supplementary Table 1.

Supplementary Table 4. Number of particles analyzed by HAADF-STEM.

| **Catalyst** | **1.2** | **2.1** | **4.4** |
| --- | --- | --- | --- |
| **Ni wt%** | 4.7 | 11.8 | 19.5 |
| **Number** | 141 | 121 | 144 |

Supplementary Figure 5. HAADF-STEM images of (a, b) Catalyst 2.1, and 4.4 nm mean particle size respectively fully oxidized NiO particles (both viewed from the [110] projection of cubic NiO), and (c, d) Catalyst 2.1 and 4.4 nm partially oxidized Ni particles.


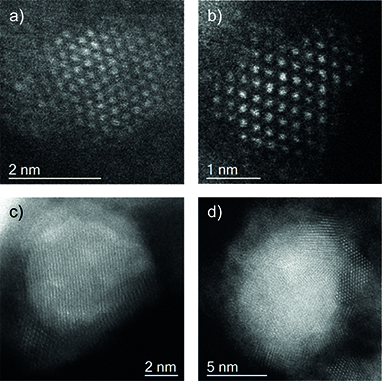


Supplementary Figure 6. Representative HAADF-STEM micrographs (lower magnification – *left column*; higher magnification – *right column)* of the Ni/SiO_2_ catalyst samples with mean particle sizes 1.2, 2.1, and 4.4 nm after their respective reduction steps, and consecutive re-oxidation under ambient conditions.


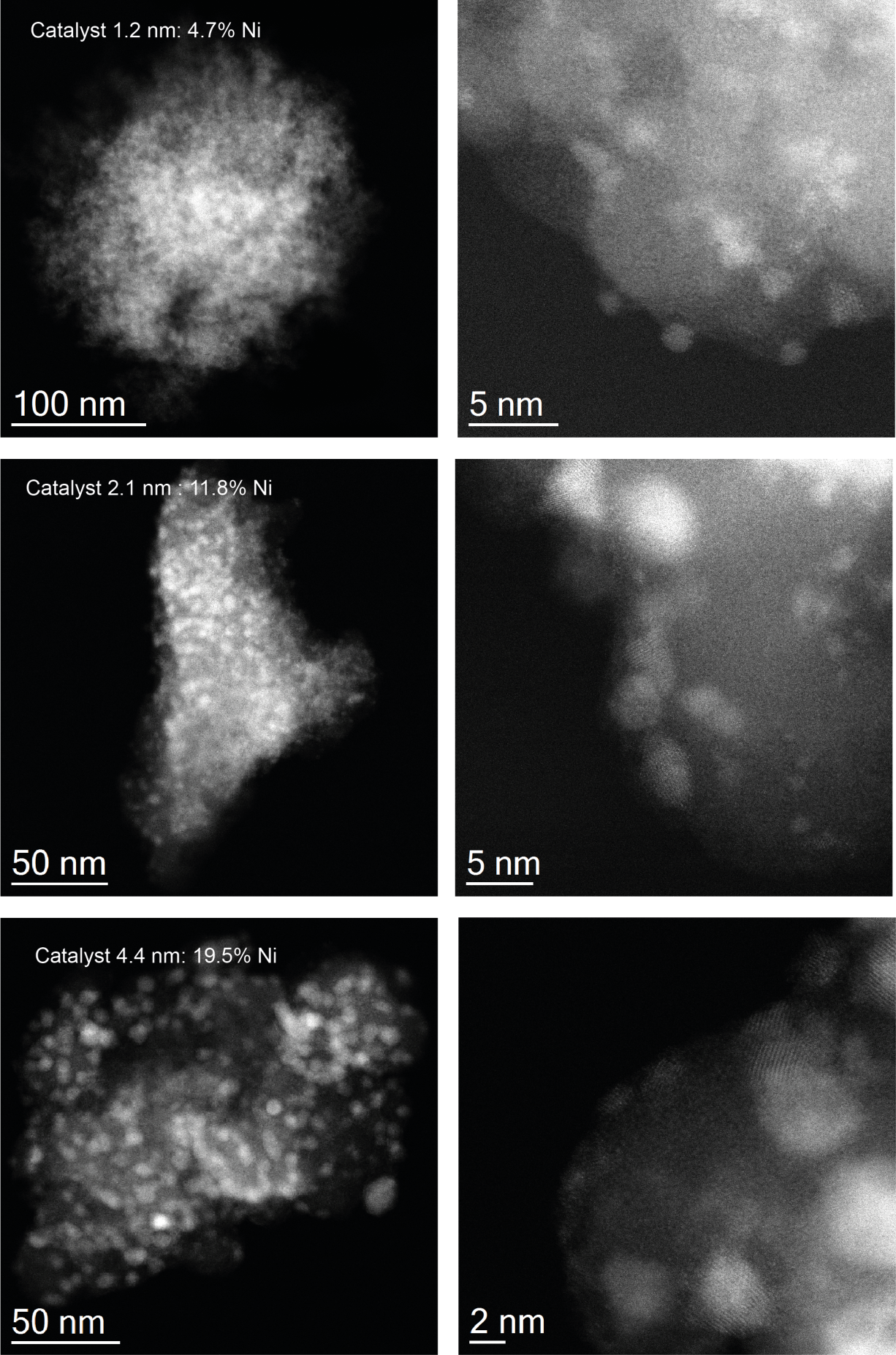


### TEM of spent (NiO) particle sizes

Supplementary Figure 7. Particle size distributions (derived from HAADF-STEM images) of the Ni/SiO_2_ catalyst samples A-H after their respective reduction steps, and consecutive re-oxidation under ambient conditions. These histograms have been corrected to take into the account (as described above) the full or partial oxidation of the Ni in catalyst sample. The particle size distributions shown here represent the *original Ni particle sizes* which are more relevant to the catalyst under actual working conditions


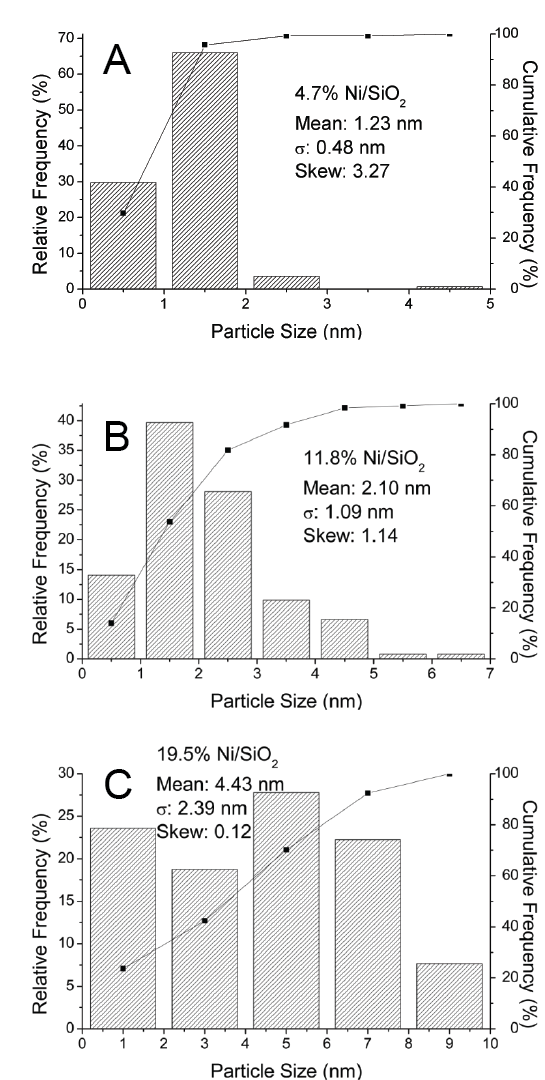

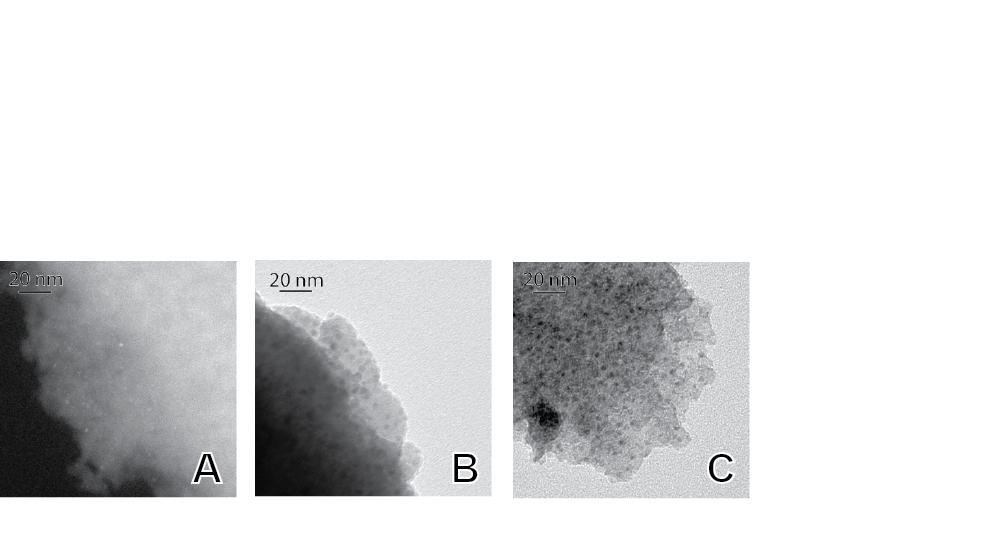


Supplementary Figure 8. TEM images the spent catalyst samples after CO_2_ hydrogenation, A-C corresponding to 1.2, 2.1, 4.4 nm, respectively.

Supplementary Figure 9. TEM derived NiO particle size distributions of the spent Ni/SiO_2_ catalysts under investigation, A-C corresponding to 1.2, 2.1, 4.4 nm, respectively.


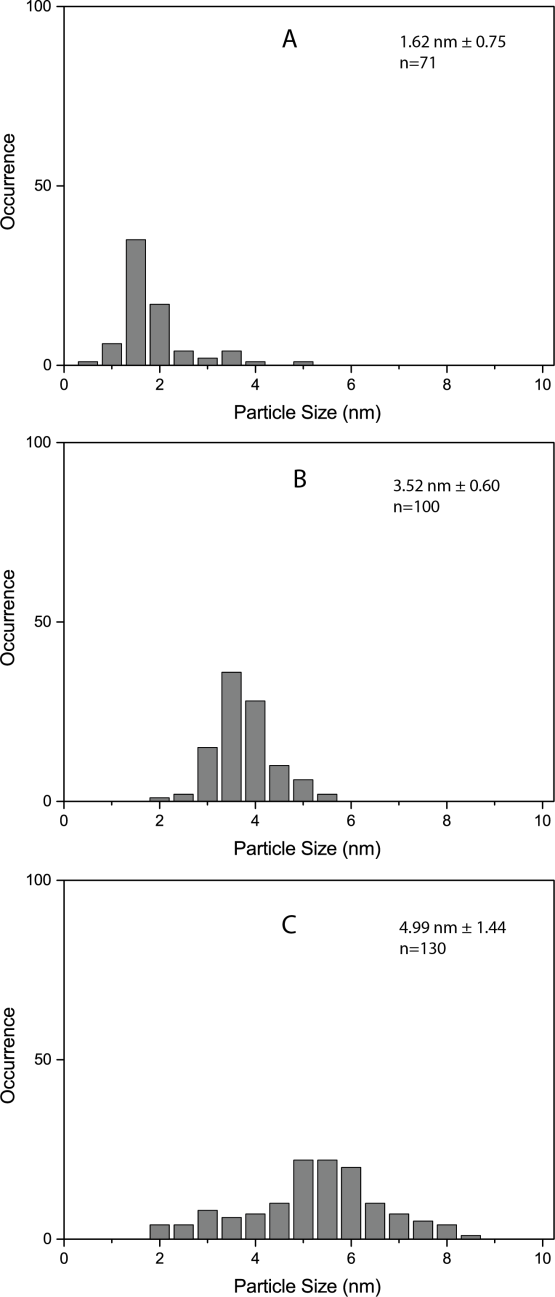


##

### Temperature Programmed Reduction

Temperature programmed reduction (TPR) was performed in a Tristar II series analyzer and is described elsewhere^9^. 550 °C was chosen as the reduction temperature as this temperature was or exceeded the middle of the second reduction peak (Ni(II) to Ni (0)) for each catalyst material. Per these results, all reduction steps in this work were ramped at 5 °C/min to, and held at the reduction temperature for 1 h.

### X-ray Absorption Spectroscopy

Operando and ex-situ (quick-) X-ray absorption spectroscopy (XAS) measurements were performed at beamline X10DA at the Paul Scherrer Institute in Villigen, Switzerland. The beamline was equipped with a Si(111) double crystal monochromator. A Ni foil was simultaneously measured as a reference with a third ionization chamber.

*Operando* quick-XAS measurements were performed in a home-built cell^9^. Powdered samples were fed into a quartz capillary and stoppered with quartz wool. Graphite ferrules hold a thermocouple in place in the gas stream, and against the quartz wool ensuring a correct temperature measurement. Gas flows were preheated to 423K.

An automated 3-way VICI valve system was custom built to remote control the gas flows in the cell, from outside the synchrotron hutch. H_2_ was attached to a single valve inlet, while N_2_ and CO_2_ together were connected to a single valve inlet. While tubing was minimalized around the cell, trace amounts of CO_2_ remained in the first N_2_ flow, and vice versa.

Operando FT-IR Spectroscopy

*Operando* Fourier transform infrared (FT-IR) spectroscopy measurements, as portrayed in Figure 1 of the main text, were performed to study reactants, intermediates and reaction products in CO_2_ hydrogenation and ethylene hydrogenation over Ni. The time-resolved *operando* FT-IR spectra were recorded to study the effect of different mean particle sizes on reaction intermediates and catalyst activity at different temperatures. The *operando* FT-IR spectroscopy measurements were carried out using a Bruker Tensor 37 FT-IR spectrometer with a DTGS detector. Spectra were recorded every 30 s for each experiment.

The FT-IR hydrogenation experiments were carried out in a Specac high-temperature high-pressure *operando* transmission IR reaction cell. To this end, the catalyst powders were pressed into wafers of approximately 16 mm in diameter, and around 0.1 mm thickness weighing between 10-15 mg. These self-supported catalyst wafers were created using a Specac Laboratory Pellet Press, a diaphragm vacuum pump and around 4 ton of pressure. Before each reaction, each catalyst was reduced at a temperature pre-determined by TPR, and reduced in-situ with a 5 °C min^-1^ temperature ramp, and a 30 min hold in a 1:1, N_2_:H_2_ flow with a total flow of 25 ml min^-1^ (both Linde, 4.9). After this in-situ reduction, the temperature of the reaction cell was brought to 100°C, and the reactants were introduced through Bronkhorst EL-FLOW Mass Flow Controllers; CO_2_ at 1.25ml min^-1^, H_2_ at 5ml min^-1^ and N_2_ to dilute at 6.25 ml min^-1^ for a total flow of 12.5 ml min^-1^. For ethylene hydrogenation reactions, the reactor was heated at a ramp rate of 5 °C min^-1^ to 150 °C or 400 °C, where the temperature was held for 90 min.

Supplementary Table 5. Vibrational assignments used for ethene hydrogenation FT-IR. An overview of the peak assignments used for CO_2_ hydrogenation FT-IR experiments can be found elsewhere^9^.

| **Wavenumber (cm^-1^)** | **Type of vibration** |
| --- | --- |
| 3015 | V_2_ C-H stretch from methane |
| 3010 | C=CH stretch |
| 2981 | V_10_ CH_3_ stretch from ethane |
| 2967 | V_10_ CH_3_ stretch |
| 2928 | C-CH_2_ asymmetric stretch |
| 2892 | CH_2_ symmetric stretch from chemisorbed ethylene |
| 2880 | CH_3_ symmetric stretch |


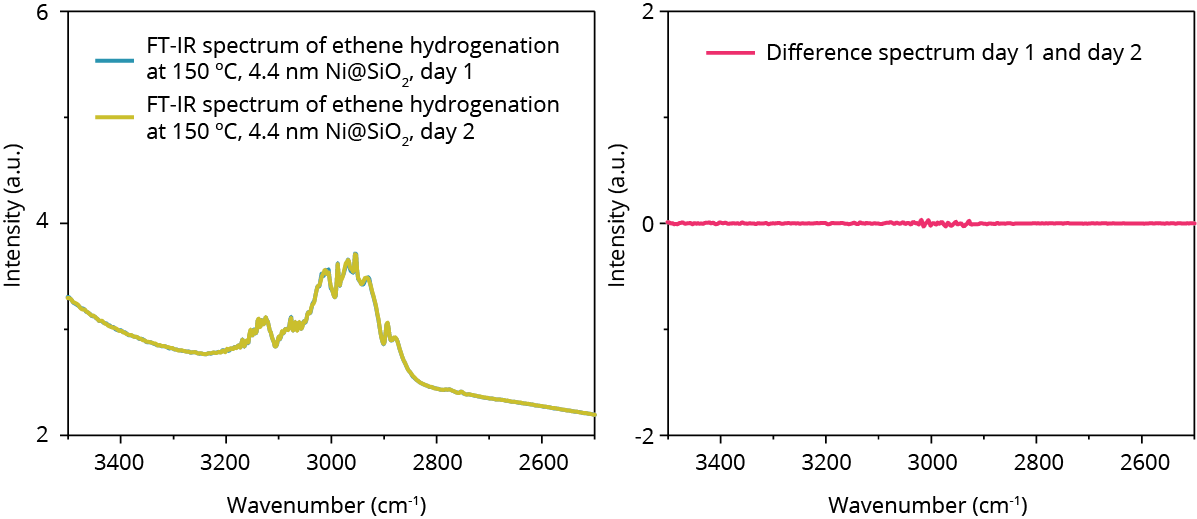


Supplementary Figure 10. Measurement error in operando FT-IR. The standard deviation in intensity is 0.0036% for repetitions of the same experiment.

### Online product analysis for operando quick-XAS and operando FT-IR and TOF determination

On-line product analysis was performed with an Interscience custom-built “ultra-fast” Global Analyzer Solutions (G.A.S) Compact GC_4.0_ gas chromatograph (GC) with a time resolution of around 10 s for lower hydrocarbons (methane, ethane, and ethene).

We wish to give some attention to two points regarding the determination and portrayal of the TOF in general, and as applied in our manuscript. Firstly, the eye guide used to show the trend in π-bond type structure sensitivity (that is, all structure sensitive reactions where the cleavage or creation of a π-bond is a rate limiting factor). We have considered the use of a quadratic function (i.e. an eventual decrease in the surface normalized activity with increasing particle size as is often drawn, and generally works for all π-type structure sensitivity) or the “hockey-stick” function (i.e. a flat TOF with increasing particle size, as is often drawn for Fischer-Tropsch synthesis, see e.g. van Helden et al.^38^) Figure 1 in the main text shows that the trend with decreasing activity for decreasing particle size holds true for all CO_2_ structure sensitivity (and a Fischer-Tropsch synthesis reaction) found in literature. This discussion is important, but not of direct significance for this work. This is why we have drawn the trend with a linear decrease with decreasing particle size as both often drawn eye guides agree in this point and it is at the heart of π-type structure sensitivity.

Secondly, we wish to show the negligible effect of particle shape on the TOF trends that we report. Supplementary Figures 11 and 12 show the area/volume ratio, and the adjusted TOF based on a hemisphere, and a hemi-truncated octahedron.

### Modulation excitation (quick-XAS and FT-IR)


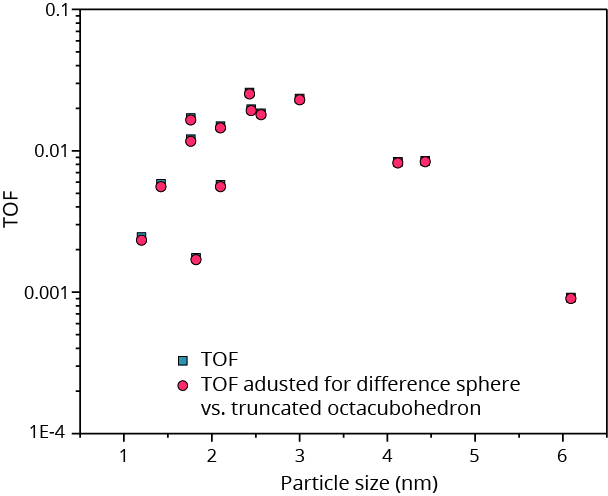


**Supplementary Figure 12.** The surface normalized activity (TOF) adjusted to accommodate two different shapes, the classical hemisphere, and the truncated octahedron. The trend reported in the main text of the manuscript remains the same.


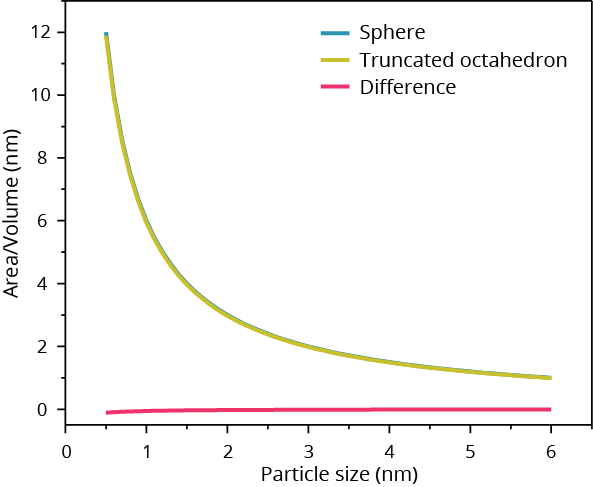


**Supplementary Figure 11.** The area divided by the volume for a sphere (as assumed in the calculation of our TOF) versus what could be the other extreme for a realistic nanoparticle shape; a truncated octahedron. By dividing the area by the volume, we correct for the steps taken in the determination of the TOF by both shapes. Supplementary Figure 12 shows the difference in TOF when we account for this shape difference.

Modulation excitation (ME) (10 cycles) and subsequent phase sensitive detection (PSD) was applied to obtain the phase angle spectra which are displayed in Figure 4 of the main text). Via consecutive stimulation and de-stimulation (e.g. with ethene, or CO­_2_ and subsequent pulses of hydrogen), and the demodulation of these changes via Supplementary Eqation 2^39^, the contribution of atoms responding to this stimulation can be singled out. Supplementary Equation 2 transforms the original signal A, a function of energy E and time t, to a phase domain rather than time. In doing so, the frequency of an external stimulation ω and the demodulation phase angle $\Phi_{k}^{PSD}$are used to cancel out all parts of the original signal that do not follow ω. This reduces the contribution of spectator species (e.g., atoms in the bulk of the nanoparticles which in no way respond to our external stimulation) and of noise. We are left with phase angle spectra, which thus, contain only spectral information of (surface) changes which modulate at the same angle as the original stimulus (pulses of reactant alternated with H_2_). Phase angle spectra (such as those displayed in Figure 4 of the main text or Supplementary Figure 18) only contain features of reversible changes.

$A_{k}^{\Phi_{k}^{PSD}}\left( E \right)= \frac{2}{T}\int_{0}^{T} A(E,t)sin(k\omega t+ \Phi_{k}^{PSD})$ Eq. 2

One is thus in principle able to separate bulk from surface, and spectator species from active ones. In essence, the added value of modulated excitation experiments is no more than other á posteriori data analysis techniques such as multivariate analysis (MVA), e.g., principal component analysis and clustering (PCA). However, in XAS, PSD is unique because one is left with the single atom scattering contribution already subtracted from your EXAFS spectrum. Furthermore, when demodulating the periodicity in the experiments, the contribution of nominally the surface with respect to the bulk is highlighted, which is interesting for e.g., studying surface changes in larger metal nanoparticles, or comparing small to large nanoparticles as we do in this study.

### Spark Ablation

Spark ablation is a technique that has only recently been developed as a commercial tool to produce nanoparticles for scientific research, mainly by the commercial availability of a tabletop setup produced by VSParticle. Spark ablation is essentially the application of a high potential difference between two metal electrodes (see Supplementary Figure 13), which ensures that some material ablates off of the electrodes due to extremely high local temperatures. These clusters then exist in the gas phase, through which they can be deposited on several media such as for example a microelectromechanical system (MEMS) reactor with which e.g., transmission electron microscopy can be performed (see Supplementary Figure 13, and section ‘In-situ HR-TEM’ for more details).

# Supplementary Note 2 – Ethylene Hydrogenation Catalysis

Supplementary Figure 13. A schematic of the procedure through which nanoclusters/particles are formed through ablation of two highly pure metallic electrodes (in this case e.g. Ni, 99.99% purity), which were deposited onto a microelectromechanical system (MEMS) reactor for in-situ studies by e.g. (scanning) transmission electron microscopy (see section In-situ HR-TEM for further details and results).


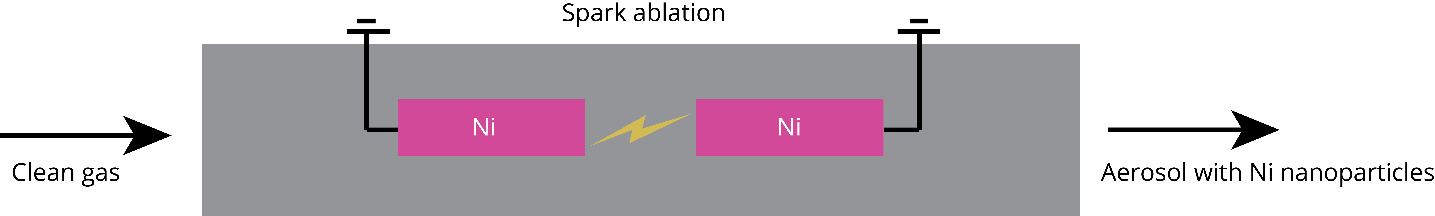


Ethylene hydrogenation over nickel is thought to be first order in hydrogen, lower order in ethylene, and with low activation energy in the order of 14 kcal ^.^ mol^-1 40^. However, at higher temperatures the activation energy decreases, and negative values have even been reported. This so-called inversion temperature occurs from 90-200°C, dependent on the pressure used. Rideal studied the hydrogenation of ethylene and concluded an optimum temperature of about 137 °C^41^. Pauls and coworkers investigated a Ni@Al_2_O_3_ catalyst at between 30 and 80°C^42^.

$C_{2}H_{4}+ H_{2}\leftrightarrow C_{2}H_{6} \Delta H= -136\frac{KJ}{mol}$ Eq. 3

The mechanism of ethylene hydrogenation is believed to follow the Horiuti-Polanyi^43^ mechanism which consists of three steps:

1. Alkene adsorption on the surface of the hydrogenated metal catalyst.
2. Hydrogen migration to the ß-carbon of the alkene with formation of a σ-bond between the metal and α-carbon (vinyl group in the case of ethylene hydrogenation).
3. Reductive elimination of the free alkane.

Furthermore, it is believed that acetylene poisons the surface of ethylene catalysts at temperatures lower than 80°C^40,42^, when hydrogenation of the alkyl or alkene intermediates is apparently not favored. However, above approximately 140°C, it is important to keep in mind that the deposition of carbonaceous species may slowly reduce the number of active sites^40^. On nickel, at adequately high temperatures (>350 °C) the fraction of these carbonaceous species which would be converted to methane should be high. Thus, the important species in this reaction are ethylene, acetylene, vinyl, methane and ethane.

Studies revealed that the active surface of Pt and Rh is covered by a monolayer of ethylidyne. This layer of ethylidyne is strongly bound, and does not hydrogenate rapidly^44,45^. Notable peaks in FT-IR for platinum are of ethylidyne at 2880 cm^-1^, of di-σ-bonded ethylene at 2910 cm^-1^, and of π-bonded ethylene^46–48^. On Pt surfaces, the direct competition of adsorption sites between ethylidyne and di-σ-bonded ethylene was shown (both adsorbing at 3-fold sites), and that pre-adsorbed ethylidyne do not affect the reaction rate. However, the availability of π-bonded species (top sites) are directly correlated with the reaction rate^48^. The explanation for the structure insensitivity of this reaction is that active intermediates are π-bonded and bind at top sites, which are available at any surface. However, it has been shown that structure insensitivity is induced in Pt clusters by opening up the reaction pathway to ethylidyne^49^.

.


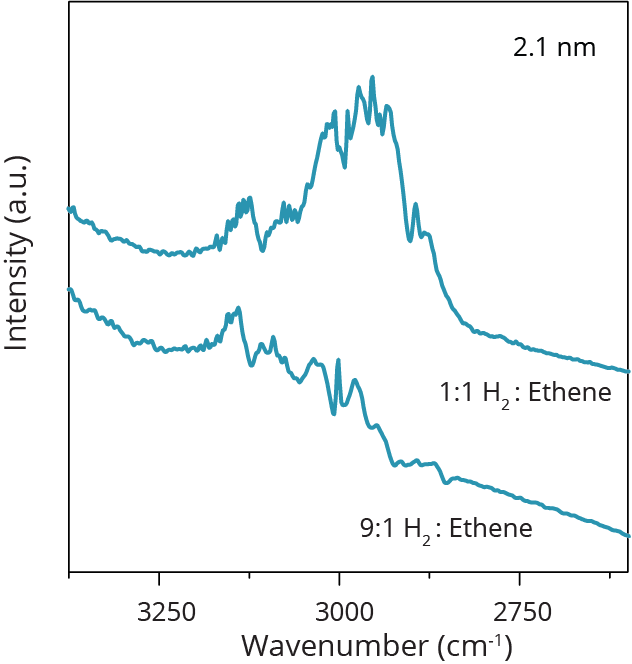


Supplementary Figure 14. Operando FT-IR of ethene hydrogenation over 2.1 nm Ni/SiO_2_ at different ratios of H_2_:ethene

# Supplementary Note 3 - CO_2_ Hydrogenation Catalysis

Classically, CO_2_ hydrogenation over Ni is considered to follow a 2-step, Langmuir-Hinshelwood type mechanism whereby first CO_2_ dissociatively adsorbs with H_2_ to form CO and H_2_O in the reverse water gas shift (RWGS) reaction. The reverse water gas shift reaction is believed to follow either of two mechanisms: firstly, the direct dissociation of CO_2_ to CO via a CO_2_^-^ ion, and secondly, a surface carbonate to formate reaction pathway. The CO is then subsequently directly hydrogenated or dissociates to atomic C_ads_ and is then hydrogenated^50,51^.

A recent study from our group shows that the formation of a surface formate intermediate in the first step of the reaction mechanism is structure sensitive. Furthermore, it suggests that the active pathway is the hydrogenation of intermediate CO_ads_, of which, in turn, the adsorption strength is particle size dependent.

${CO}_{2}+ H_{2}\leftrightarrow CH_{4}+ H_{2}O \Delta H= -165\frac{KJ}{mol}$ Eq. 4

The most important detectable species in this reaction are thus; CO_ads_, formate, gaseous CO_2_, and methane.

# Supplementary Discussion - X-ray Absorption Spectroscopy

To determine what may be causing the perturbation onset by ethene hydrogenation (Figure 1F in the main text), Supplementary Figure 15 shows the Fourier transform of the extended X-ray absorption fine structure (EXAFS) recorded under pure H_2_ and in pure ethene atmosphere, respectively. While the effect of thermal and catalytic restructuring may play a role in reducing the local order, it is clearly visible that a shoulder appears in an ethene environment at a scattering distance shorter than the Ni-Ni distance. As there is no oxygen in the system, we tentatively attribute this contribution to the (surface) formation of Ni-C bonds. Multiple groups have shown the formation of carbide phases in and on different (noble) metals^52–54^, the EXAFS in Supplementary Figure 15 hints toward the formation of such Ni carbide as well. This carbide may be in the surface layers, or the bulk, see the main text.


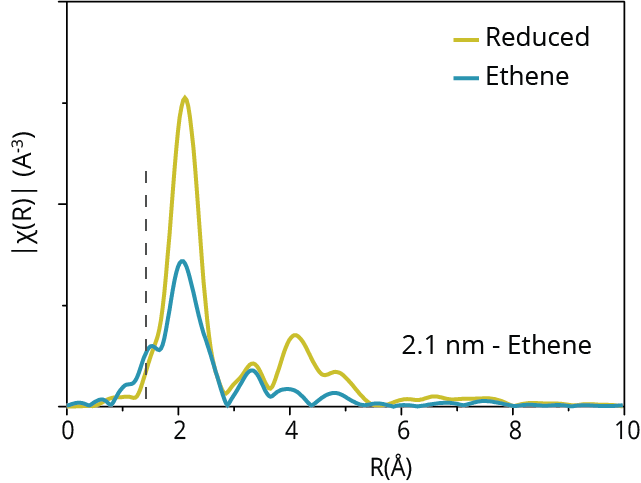


Supplementary Figure 15. XAS spectrum after reduction, and exposure to ethene at reaction conditions (150 °C. The dotted line indicates a proposed C scattering path.

Supplementary Figure 16. a) Single X-ray absorption spectrum (recorded at 0.16 s). b) R-space of single X-ray absorption spectrum of the Ni/SiO_2_ catalyst sample with 4.4 nm mean Ni particle size recorded at 0.16 s speed.


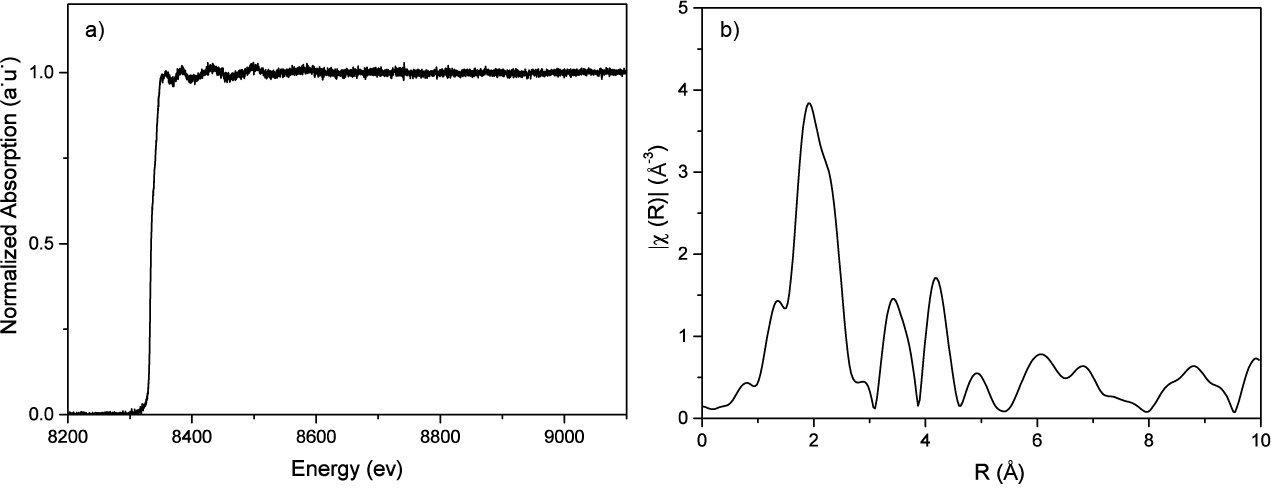


Supplementary Figure 17. Top, X-ray absorption spectra of Ni/SiO_2_ catalyst sample with 4.4 nm mean Ni particle size binned 200x in time, during CO_2_ (left), ethene (right) and H_2_ pulse, i.e. 30 s time resolution, and a zoom-in of the K-edge region. Bottom, difference spectrum of the 200x binned spectra shown in the top panel. For CO_2_ (left), and ethene (right) versus H_2_ pulse experiment.


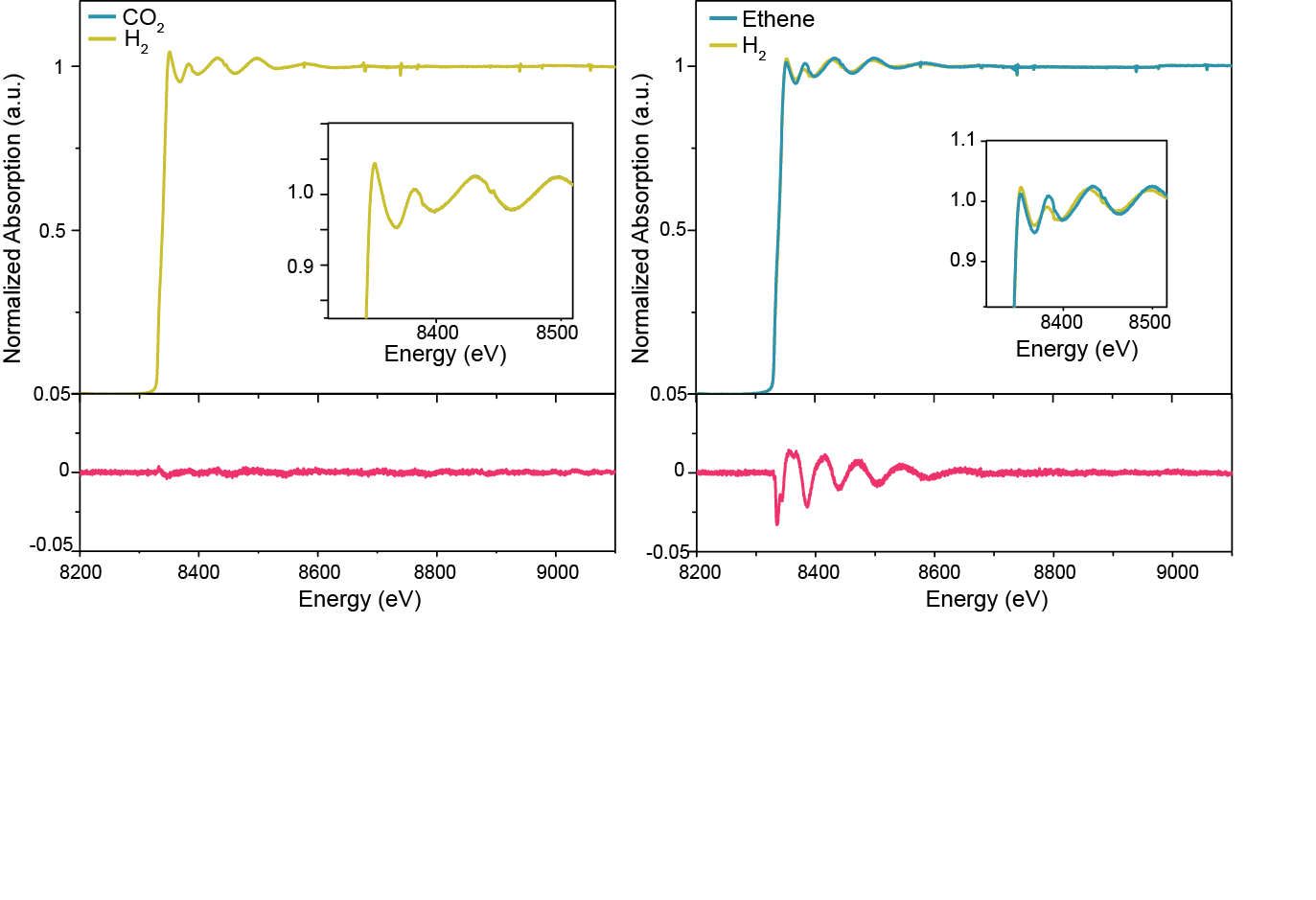


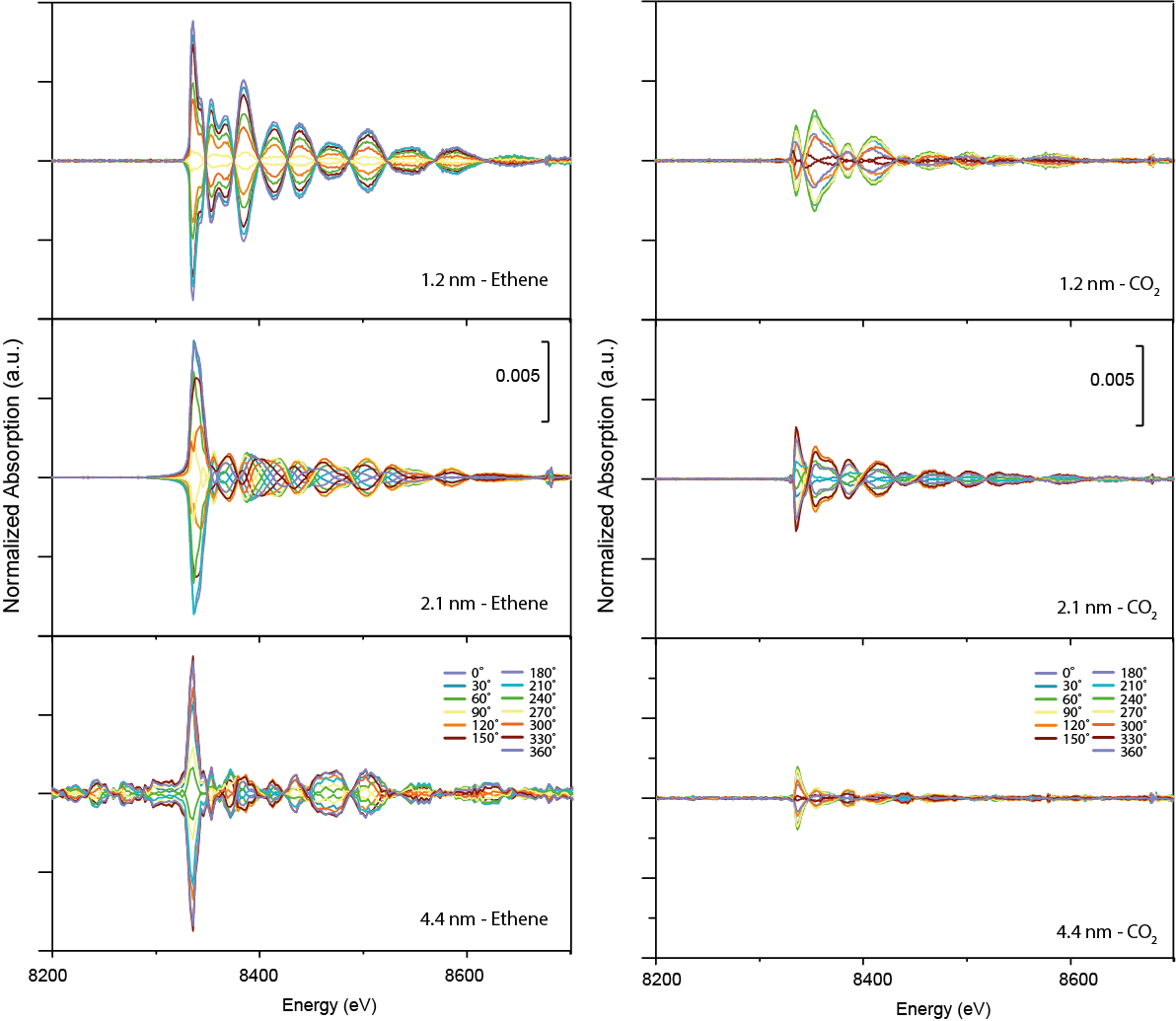


Supplementary Figure 18. Phase angle spectra of ethene pulsed experiments (left) at 150 °C, and CO_2_ (right) pulsed experiments at 400 °C for 3 different mean Ni particle size.


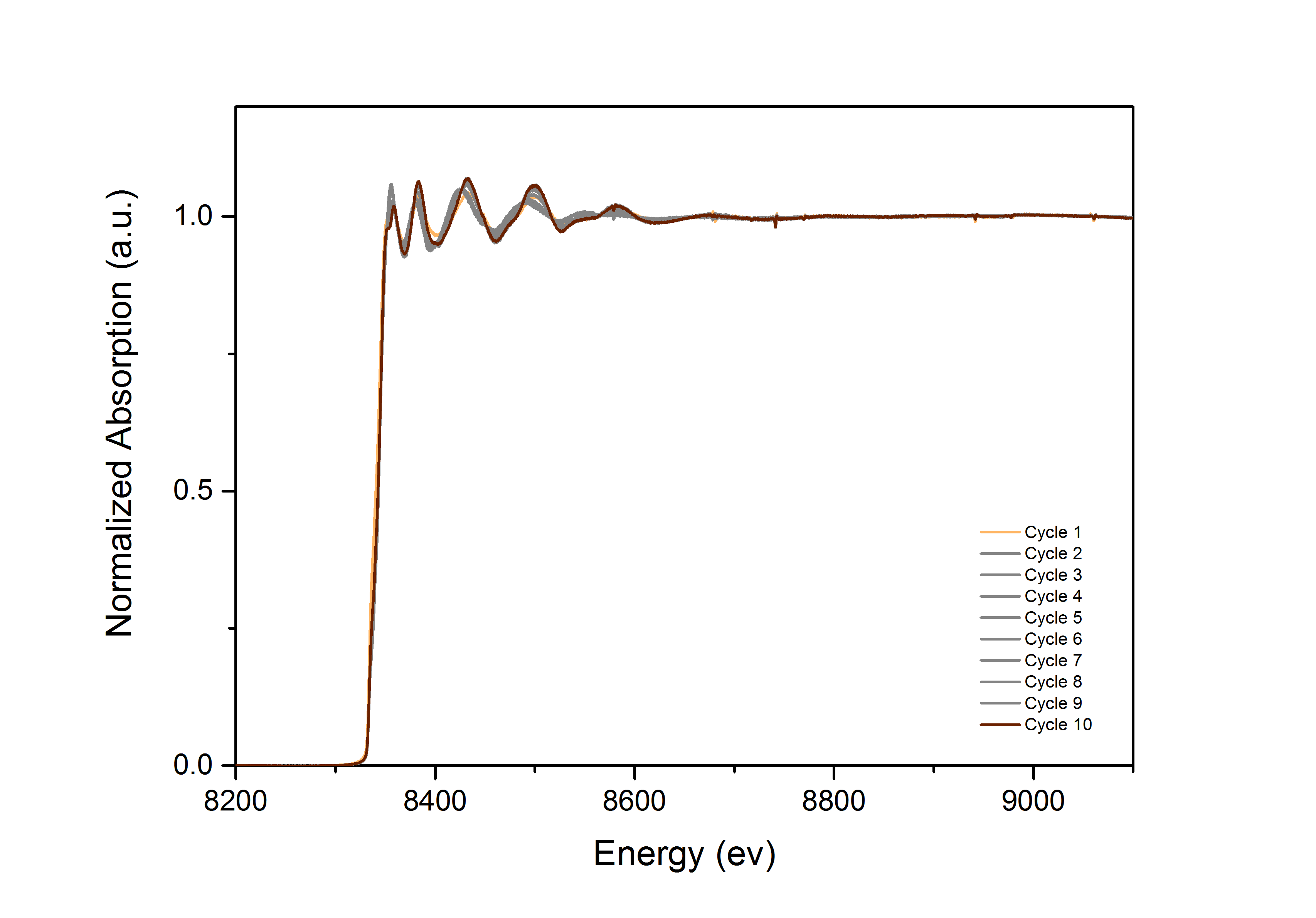


Supplementary Figure 19. X-ray absorption spectra (200x binned in time) during the 10 cycles of the ethene modulated excitation experiment with the 2.1 nm mean Ni nanoparticle size to show the pulse-induced changes over multiple cycles.


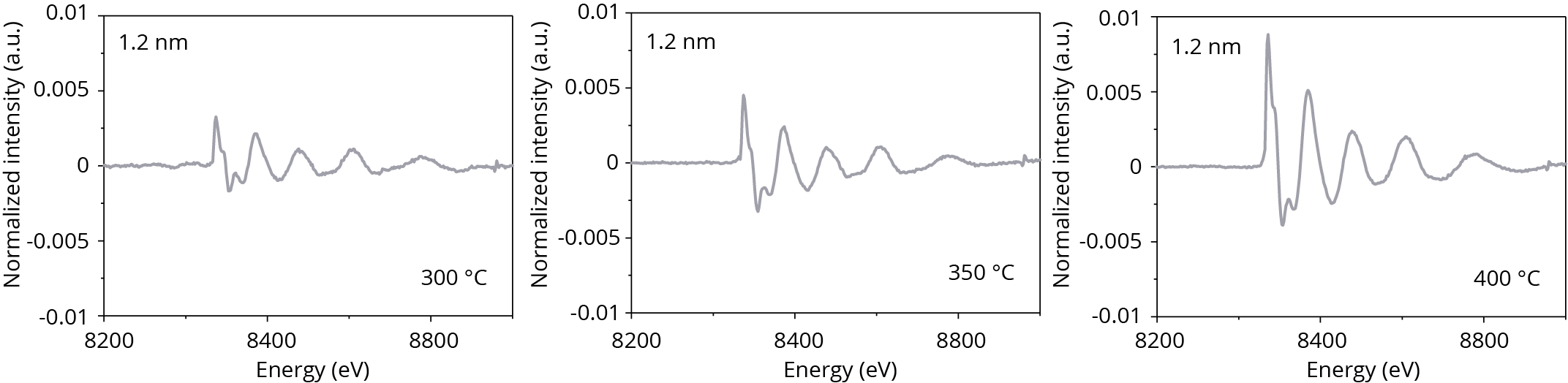


Supplementary Figure 20. Temperature dependent restructuring data. The figures show the difference spectra of the ethene-induced restructuring events occurring at 300, 350, and 400 °C.


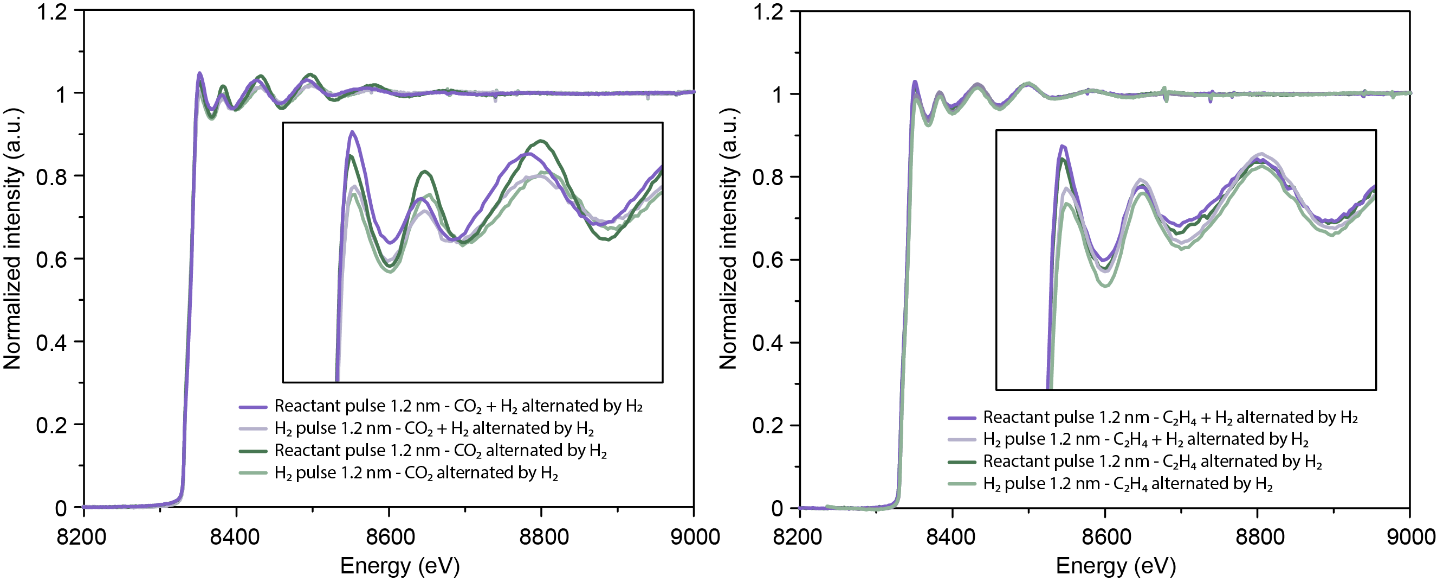


Supplementary Figure 21. Average quick X-ray absorption spectra during each pulse of either reactant, or a hydrogen pulse, serving to show very similar trends whether catalysis (reactant + H_2_) or reactant pulsing was offset by H_2_ pulses.


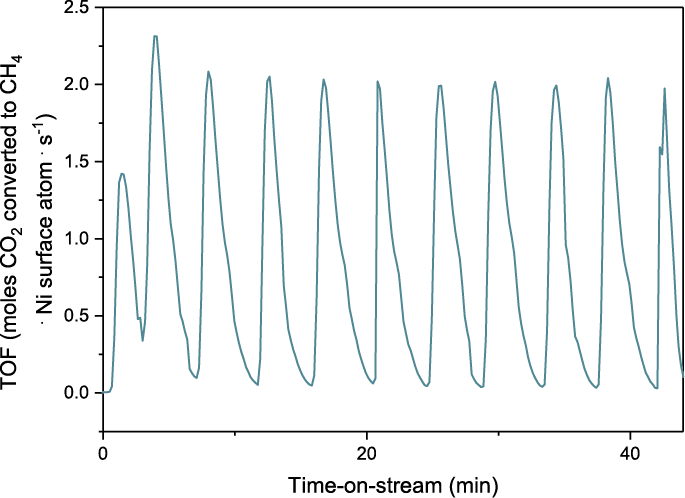


Supplementary Figure 22. TOF values as measured for the 2.1 nm Ni/SiO_2_ during operando Q-XAS measurements. These data were measured by GC and show that the order of magnitude in the TOF values during these measurements is the same as previously reported^1^.

A quick-XAS experiment of approximately 45 min collected at 10 Hz results in approximately 60.000 spectra (spectra are collected in both the up and down movement of the monochromator). To determine the parameters with which the entire set of spectra was fit, two outlier spectra were produced by averaging the equivalent of 33 seconds of acquisition during the first H_2_ pulse (fresh) and during the first ethene pulse. An appropriate fitting model was chosen first by varying all fitting parameters in the fits (including the coordination numbers), except for σ ^2^ which was determined for every experiment with the appropriate Ni foil reference. While this is the most objective fitting model, the coordination numbers are too close within one experiment (one sample with varying conditions), *i.e.*, within error bars, to be discriminated reliably. Because these results were obtained for the outliers, the contrast in the remaining experimental data will be much smaller. The biggest changes detected in this fitting model are in the σ^2^ values which should then be interpreted as primarily caused by the surface restructuring that is, evidently, adsorbate-mediated^55^. Quantitative analysis of the σ^2^ can be done in terms of the surface-centric bond length disorder.

Supplementary Figure 23 shows the R-space EXAFS of the catalysts under study after their reduction procedure. The intensity of the amplitude of the first Ni-Ni scattering pathway for the different Ni@SiO_2_ catalyst samples correlates to the mean measured nanoparticle size by HAADF-STEM, the coordination numbers after reduction from the first-shell coordination fit are very close together indicating for the 4.4 nm particle size a smaller particle size than measured with HAADF-STEM and the other characterization techniques. Supplementary Tables 4 and 5 show the fitting model for the two outliers for the ethene-pulsed experiments shown in Figure 2 of the main text, to produce Figure 3 of the main text. Supplementary Figure 24 shows a full data set, and the result of the first-shell coordination fit is shown in Supplementary Figure 25.


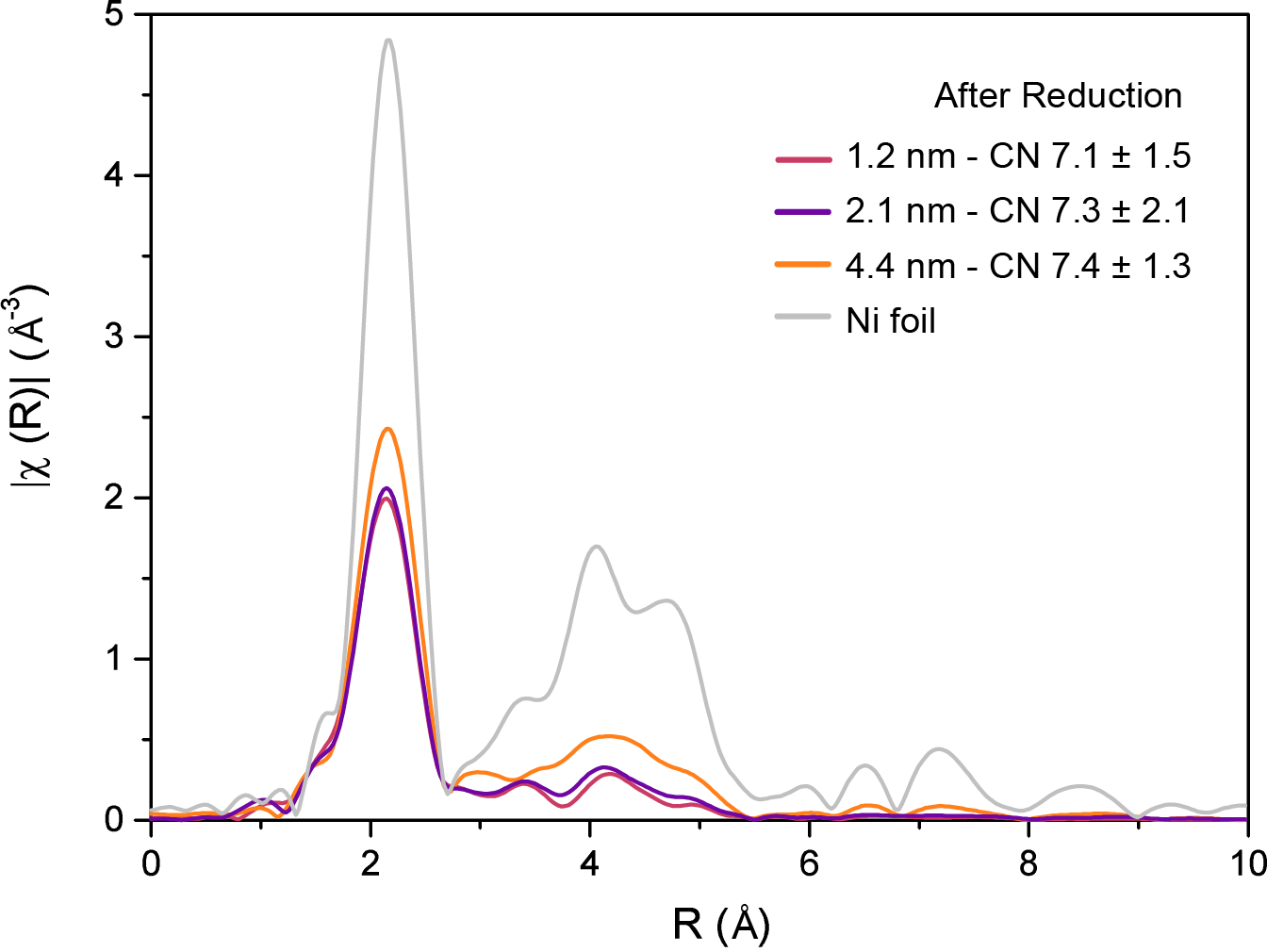


Supplementary Figure 23. X-ray absorption spectra of the Ni@SiO_2_ nanoparticles after reduction in H_2,_ and the corresponding coordination numbers (CN) from a first-shell coordination fit.

**Supplementary Table 6.** Fitting parameters obtained by fitting two outliers (in H_2_ and CO_2_) for each experiment, which were applied to batch fitting by LARCH for approximately 70.000 spectra per experiment.

| 1.2 nm | | |
| --- | --- | --- |
| N = 7.5 | | |
|  | H_2_ | CO_2_ |
| ΔE0 | -6.6 ± 2.3 eV | -10.4 ± 2.5 eV |
| ΔR | -0.034 ± 0.016 Ǻ | -0.061 ± 0.017 Ǻ |
| σ^2^ | 0.013 ± 0.0018 Ǻ^2^ | 0.0139 ± 0.0018 Ǻ^2^ |
| R(Ni-Ni) | 2.455 Ǻ | 2.428 Ǻ |
| Reduced chi-square | 13.5 |  |
| R-factor | 0.0385 |  |

| 2.1 nm | | |
| --- | --- | --- |
| N = 6.0 | | |
|  | H_2_ | CO_2_ |
| ΔE0 | -6.7 ± 0.8 eV | -6.8 ± 0.8 eV |
| ΔR | -0.031 ± 0.005 Ǻ | 0.0059 ± 0.06 Ǻ |
| σ^2^ | 0.0131 ± 0.0006 Ǻ^2^ | 0.014 ± 0.0064 Ǻ^2^ |
| R(Ni-Ni) | 2.46 Ǻ | 2.46 Ǻ |
| Reduced chi-square | 11.9 | |
| R-factor | 0.00496 | |

| 4.4 nm | | | |
| --- | --- | --- | --- |
| N = 6.7 | | | |
|  | H_2_ | | CO_2_ |
| ΔE0 | -7.952 ± 0.94 eV | -9.812 ± 2.22 eV | |
| ΔR | -0.0335 ± 0.007 Ǻ | -0.0335 ± 0.007 Ǻ | |
| σ^2^ | 0.013 ± 0.0008 Ǻ^2^ | 0.0121 ± 0.001 Ǻ^2^ | |
| R(Ni-Ni) | 2.455 ± 0.007 Ǻ | 2.437 ± 0.007 Ǻ | |
| Reduced chi-square | 6.7 | | |
| R-factor | 0.017 | | |

**Supplementary Table 7.** Fitting parameters obtained by fitting two outliers (H_2_ and ethene) for each experiment, which were applied to batch fitting by LARCH for approximately 70.000 spectra per experiment.

| 1.2 nm | | |
| --- | --- | --- |
| N = 7.5 | | |
|  | H_2_ | Ethene |
| ΔE0 | -6.8 ± 1.0 eV | -9.7 ± 1.7 eV |
| ΔR | -0.023 ± 0.007 Ǻ | -0.009 ± 0.012 Ǻ |
| σ^2^ | 0.0104 ± 0.0008 Ǻ^2^ | 0.0153 ± 0.0011 Ǻ^2^ |
| R(Ni-Ni) | 2.466 Ǻ | 2.480 Ǻ |
| Reduced chi-square | 52.03 |  |
| R-factor | 0.0103 |  |

| 2.1 nm | | |
| --- | --- | --- |
| N = 6.0 | | |
|  | H_2_ | Ethene |
| ΔE0 | -6.6652 ± 1.97 eV | -9.539 ± 3.061 eV |
| ΔR | -0.0221 ± 0.015 Ǻ | 0.0286 ± 0.025 Ǻ |
| σ^2^ | 0.01513 ± 0.002 Ǻ^2^ | 0.0193 ± 0.0024 Ǻ^2^ |
| R(Ni-Ni) | 2.4669 Ǻ | 2.5176 Ǻ |
| Reduced chi-square | 113.75 | |
| R-factor | 0.0512 | |

| 4.4 nm | | | |
| --- | --- | --- | --- |
| N = 6.7 | | | |
|  | H_2_ | Ethene | |
| ΔE0 | -6.0979 ± 1.21 eV | | -7.994 ± 1.29 eV |
| ΔR | -0.0222 ± 0.008 Ǻ | | -0.0134 ± 0.009 Ǻ |
| σ^2^ | 0.00979 ± 0.0008 Ǻ^2^ | | 0.0131 ± 0.0009 Ǻ^2^ |
| R(Ni-Ni) | 2.4755 ± 0.008 Ǻ | | 2.4668 ± 0.009 Ǻ |
| Reduced chi-square | 51.85 | | |
| R-factor | 0.012 | | |

Supplementary Figure 24. Example of the data analysis for quick-X-ray absorption spectroscopy. Here all Fourier-transformed extended X-ray absorption fine structure data (EXAFS, R-space) acquired for the 4.4 nm Ni particle size during the ethene experiment are shown transposed with the first-shell-coordination fit at the top of the image. All approximately 30.000 spectra are shown from a side angle at the bottom left, and the first-shell-coordination fit is plotted at the bottom right.


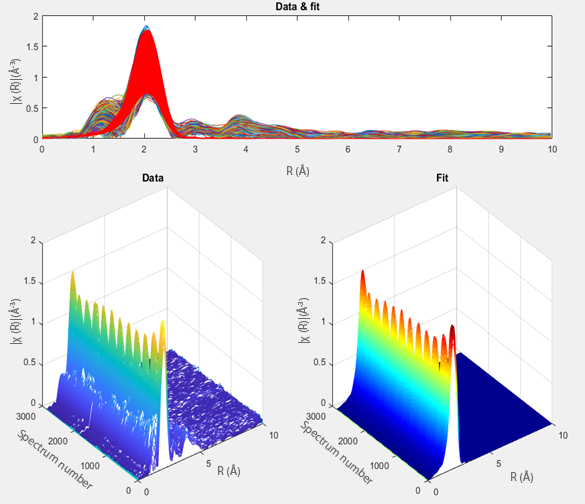


Supplementary Figure 25. Fitting parameters from the ethene-pulsed quick-X-ray absorption experiment shown in Supplementary Figure 24 plotted against the spectrum number.


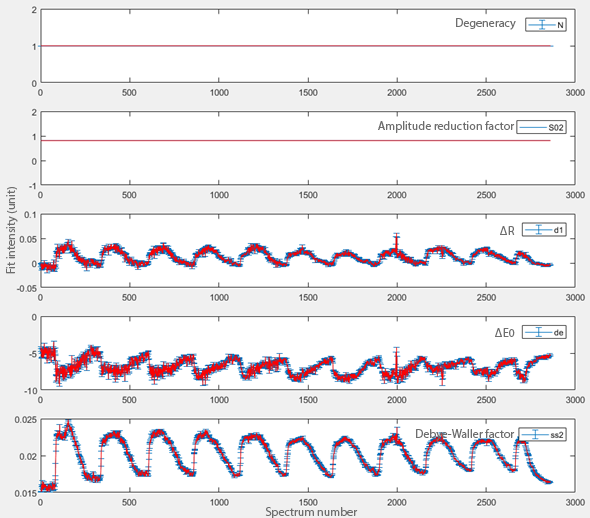


**
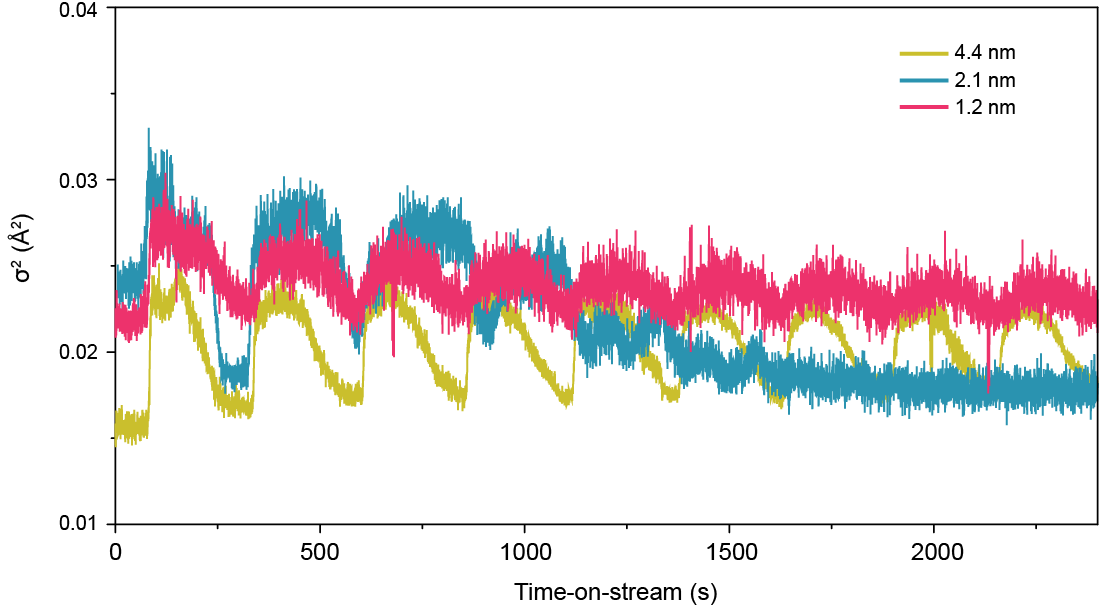
**

**Supplementary Figure 26.** Batch analysis of X-ray absorption spectra of ethene-pulsed modulated excitation experiments. For each particle size, 50.000-70.000 X-ray absorption spectra were individually fit, fixing the coordination number but varying σ^2^_._ The σ^2^ as plotted here should be interpreted as primarily caused by the surface restructuring that is, evidently, adsorbate-mediated.

**
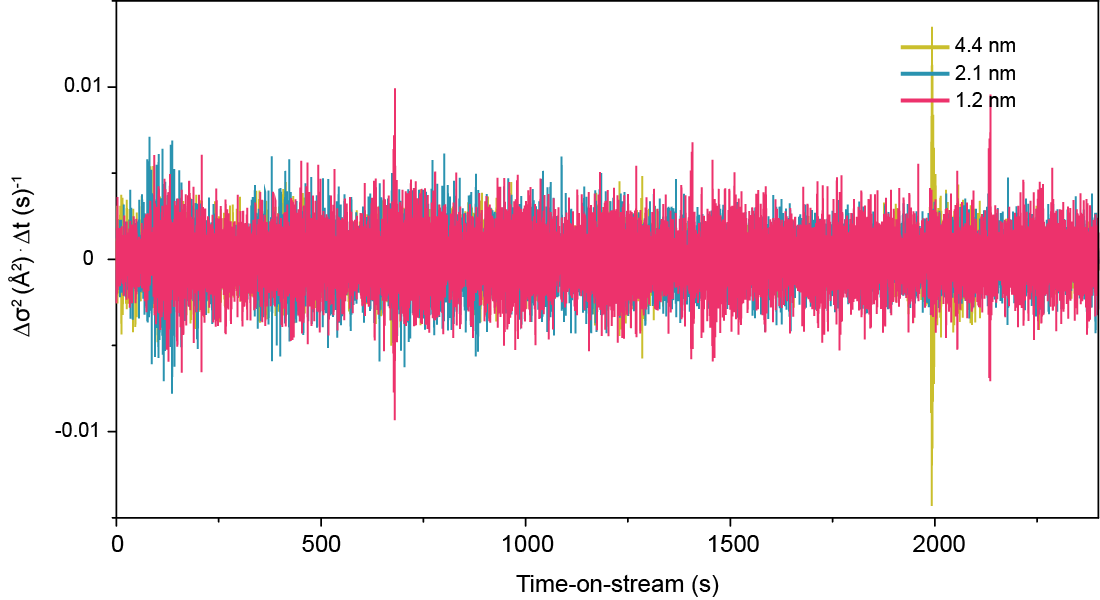
**

**Supplementary Figure 27.** From Supplementary Figure 26, the change in σ^2^ from fitting of the X-ray absorption spectra relative to time, plotted against time in the batch analysis of ethene-pulsed modulated excitation experiments.


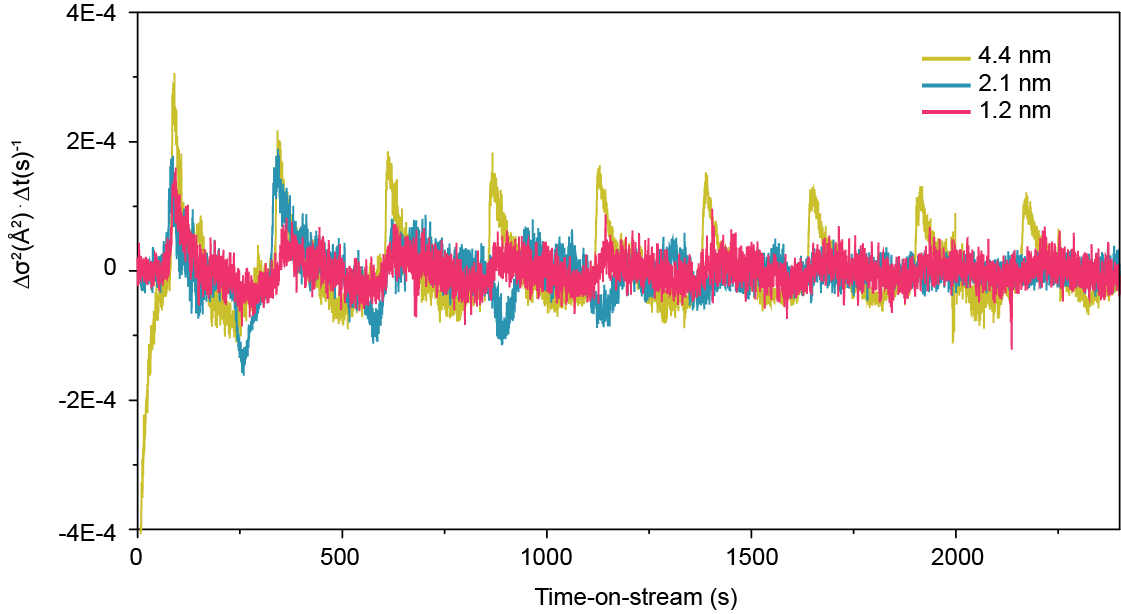


**Supplementary Figure 28.** From Supplementary Figure 27, smoothed change in σ^2^ from fitting of the X-ray absorption spectra relative to time (exponential smoothing factor α = 0.99), plotted against time in the batch analysis of ethene-pulsed modulated excitation experiments.

# Supplementary Discussion - **In-situ HR-TEM**


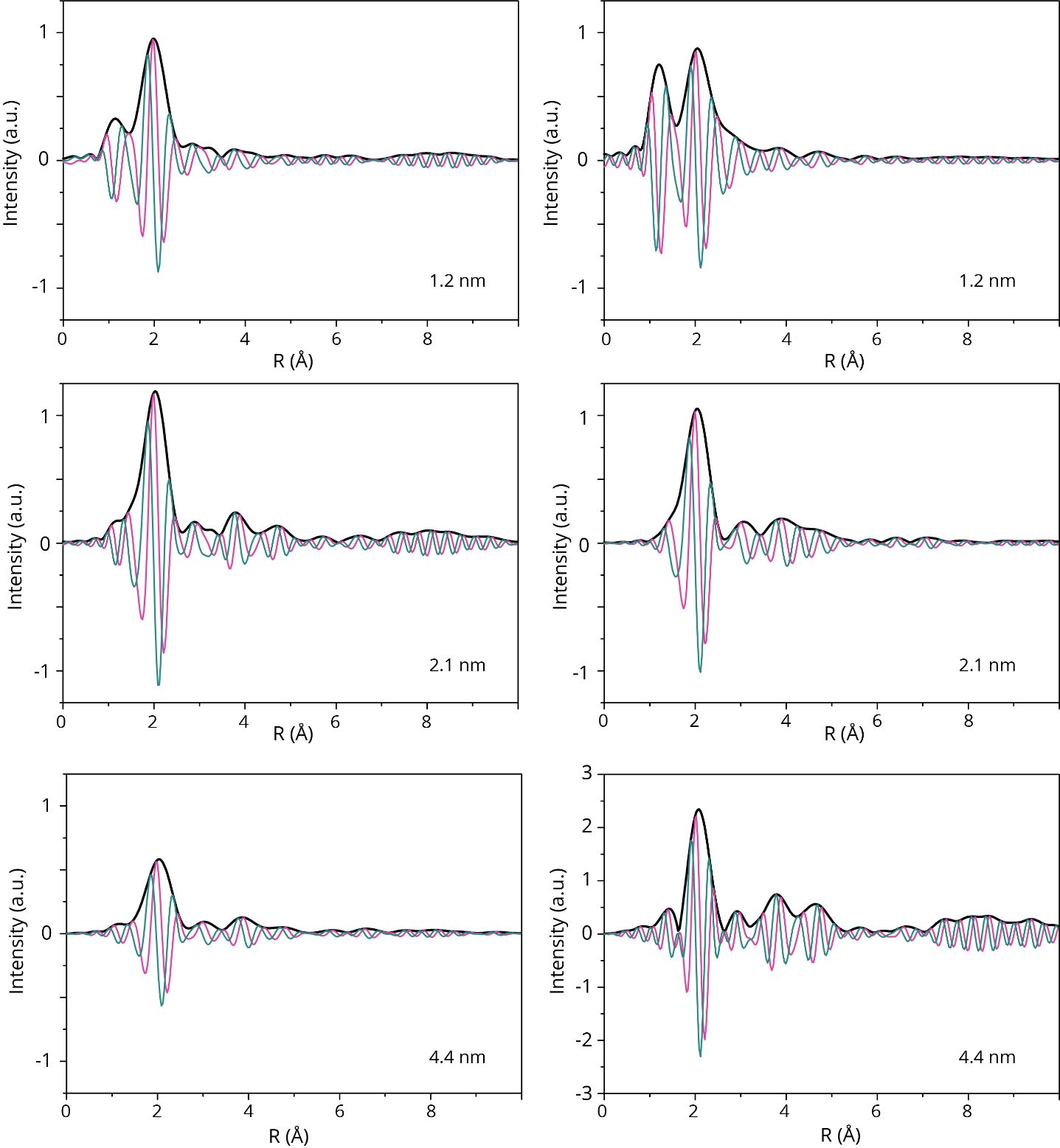


Supplementary Figure 29. The average Fourier transform X-ray absorption spectrum (and real and imaginary part) of the operando time-resolved quick-EXAFS experiment in Figure 2 of the main text.

In-situ HR-TEM measurements were performed in a 300 kV aberration-corrected STEM/EELS FEI Titan S microscope, at Oak Ridge National Laboratory in the United States. Samples were prepared by flowing a Ni nanoparticle-containing aerosol (see Supplementary Figure 13) directly onto the masked Si_x_N_y_ windows of a Protochips Atmosphere MEMS-based reactor system. These reactors contain a SiC thin film heating element, and a gas in- and outlet allowing for the measurement of the deposited nanoparticles in various gaseous atmospheres and at elevated temperatures. Before each experiment, a reduction procedure was performed in pure hydrogen at 450 °C for 1 h. It was noted that if this reduction procedure was not performed, a significant amount of carbon deposit would occur upon examination with the electron microscope. Furthermore, the particles would move around when measuring. Thus, this reduction procedure was performed before any examination by electron microscopy.

Supplementary Figure 30. Schematic showing the basic principles of the microelectric mechanical reactor, and the Si_X_Ny windows through which Ni nanoparticles can be examined by HR-TEM under elevated temperatures and under different gaseous environments.


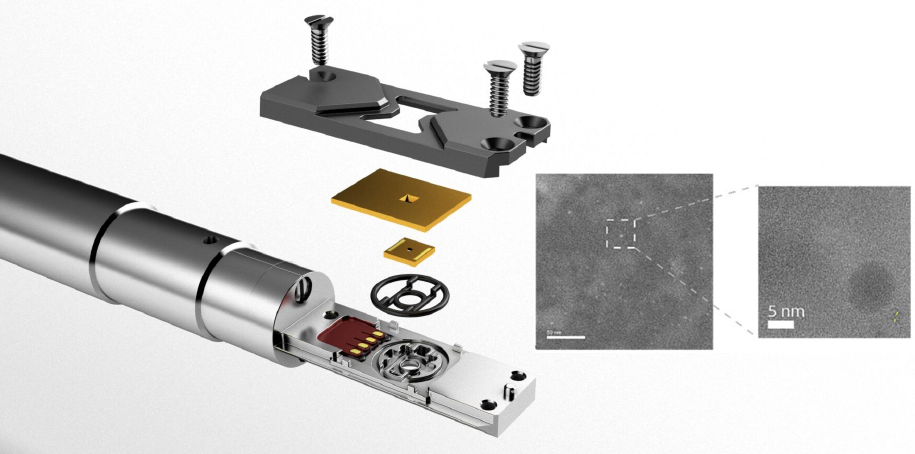


Subsequently, the cell was cooled down to either 150 or 400 °C, and ethylene, or CO_2_ was flown, respectively. Thereafter, hydrogen was flown at the same reaction temperature. The grain boundaries (Supplementary Figure 31), and lattice expansion or contraction (Supplementary Figure 33) of several nanoparticles could thereby be measured.


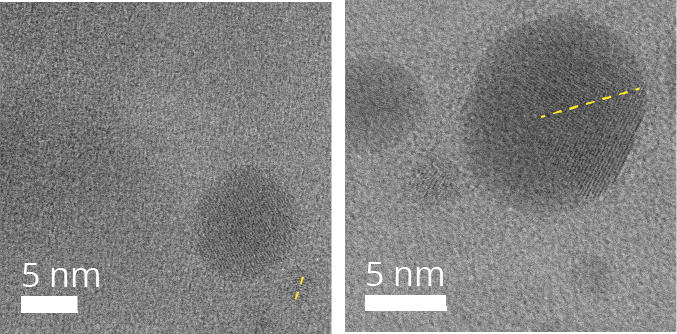


Supplementary Figure 31. In-situ bright-field scanning transmission electron microscopy images of Ni nanoparticles under N_2_, after exposure to H_2_, with examples of observed grain boundaries indicated by yellow dotted lines, images recorded at 400 °C.





Supplementary Figure 32. In-situ annular dark-field scanning transmission electron microscopy image of Ni nanoparticles under N_2_, after exposure to H_2_environment at 150ºC, before the ethene experiments shown in Figure 3 of the main text.

Supplementary Figure 33. In-situ bright-field scanning transmission electron microscopy images of Ni nanoparticles under N_2_ and the corresponding fast Fourier transform (FFT) of the areas indicated by a yellow box for lattice spacing measurements.


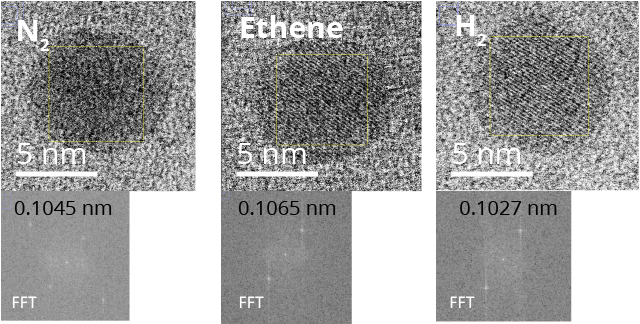


# Supplementary Note 4 – Estimate of the Diffusion Coefficient of C through Ni Using the Time-Dependent Reaction Data





N_2_





CO_2_

0.088 nm

0.092 nm

0.092 nm

0.093 nm

Supplementary Figure 34. In-situ bright field scanning transmission electron microscopy images of Ni nanoparticles under N_2_ and under CO_2_ environment at 400ºC, and corresponding fast Fourier transform (FFT) measurements of the lattice spacing.

5 nm

5 nm

5 nm

Definition of the EXAFS Debye Waller factor (DWF) is^56^:

$DWF=e^{-2\sigma^{2}k^{2}}$ Eq. 5

Where $\sigma^{2}$ is the mean-square relative displacement (MSRD) of absorber and backscatterer atoms:

$\sigma^{2}=\frac{1}{N}\sum_{i} \left( R_{i}-\bar{R} \right)^{2}$ Eq. 6

where $R_{i}$ and $\bar{R}$ are the individual paid lengths and their mean values, respectively. From Einstein’s diffusion equation it can be derived that:

$\sigma^{2}=6Dt$ Eq. 7

Where *D* is the diffusion coefficient of C in Ni and *t* is time. The ratio ${\sigma^{2}}/t$ is shown in Supplementary Figure 28 and equal to the value of $6D$, and thus the value of $D$ expressed in Å^2^ s^-1^ can be obtained.

$D$ of C in Ni is equal to^57^:

$D=e^{0.909-{20200}/T}$ Eq. 8

where *T* is the temperature in Kelvin and *D* is in cm^2^ s^-1^, which at 150 °C (423 K) is equal to 4.60 ^.^ 10^-21^ cm^2^ s^-1^.

**Supplementary Table 8.** Calculated carbon diffusion coefficients for different Ni particle sizes supported on SiO_2_.

| Ni sample | Calculated carbon diffusion coefficient (cm^2^ s^-1^) |
| --- | --- |
| 1.2 nm | 2.67 ^.^ 10^-21^ |
| 2.1 nm | 2.97 ^.^ 10^-21^ |
| 4.4 nm | 5.10 ^.^ 10^-21^ |

This is comparable to $D$ of C in Ni, equal to^57^: $D=e^{0.909-{20200}/T}$, where *T* is the temperature in Kelvin and *D* is in cm^2^ s^-1^, which at 150 °C (423 K) is equal to 4.60 ^.^ 10^-21^ cm^2^ s^-1^.

Such diffusion values indicate that, in a time in the order of 10 seconds (in which we observe the variation of $\sigma^{2}$), the nuclear mean square displacement of C is in the order of 1x10 Å^-2^. Since this is much smaller than a Ni-Ni distance (2.5 Å), carbon should only occupy surface sites in our case.

Nonetheless, as Supplementary Figure 28 shows, the 1.2 nm NPs have the smallest change in the $\sigma^{2}$ values, and the 4.4 nm the greatest, with a ratio of $\frac{{\Delta\sigma}^{2}(1.2)}{{\Delta\sigma}^{2}(4.4)}=0.76$. This is unexpected since the ratio of the surface sites in 1.2 nm and 4.4 nm nanoparticles is $\frac{x_{surf}(1.2)}{x_{surf}(4.4)}\approx\frac{0.5}{0.15}=3.3$, indicating that either the smaller nanoparticles surface is not entirely restructured or that the bigger nanoparticles are restructuring also (partially) in their bulk. Notably, carbon diffusion in Ni was reported to be highly anisotropic due to the difference in energy barrier for migration parallel to grain boundary (E_a_= 42-74 kJ mol^-1^) versus lattice diffusion (E_a_=160 kJ mol^-1^)^58^. Such a difference in activation energy at 150 °C results in about 10^10^ times faster kinetics, so that C migration along boundaries may happen in the order of seconds. We therefore argue that the ${\Delta\sigma}^{2}$ratio observed for the nanoparticles can be explained by carbon can diffusion in the bigger nanoparticles along grain boundaries, which were observed by HRTEM (see Supplementary Figure 24).

# Supplementary Note 5 – Estimate of the Volume Fraction of C Intercalation Sites in Ni Nanoparticles from their Disorder Parameters Measured by EXAFS

As Supplementary Figure 28 shows, the 1.2 nm NPs have the smallest change in the $\sigma^{2}$ values, and the 4.4 – the greatest. By using these data and a simple model the fraction of the sites available for C intercalation into Ni nanoparticles can be estimated, as shown below. From the definition of the EXAFS mean-square relative displacement:

$\sigma^{2}=\frac{1}{N}\sum_{i} \left( R_{i}-\bar{R} \right)^{2}= \sigma_{T}^{2}+\sigma_{s}^{2}$ Eq. 9

where $R_{i}$ and $\bar{R}$ are the individual paid lengths and their mean values, respectively, and the last two terms are the thermal (dynamic) and configurational (static) factors, respectively. We express the mean square relative displacement (MSRD) values for the 1.2 and 4.4 nm nanoparticles as a function of the volume fraction of sites available for C intercalation (x) during and after the ethene pulse as follows:

1.2 nm: ${\Delta\sigma}^{2}=\frac{1}{N}\left[ \sum_{i} \left( R_{i}-\bar{R} \right)^{2} \right]_{on}-\frac{1}{N}\left[ \sum_{i} \left( R_{i}-\bar{R} \right)^{2} \right]_{off}$=$\sigma_{T}^{2}\left( 1-x \right)+\sigma_{T}^{2}x+\sigma_{s}^{2}x-\sigma_{T}^{2}=\sigma_{s}^{2}x$ Eq. 10

4.4 nm: ${\Delta\sigma}^{2}=\frac{1}{N}\left[ \sum_{i} \left( R_{i}-\bar{R} \right)^{2} \right]_{on}-\frac{1}{N}\left[ \sum_{i} \left( R_{i}-\bar{R} \right)^{2} \right]_{off}$=$\sigma_{T}^{2}+\sigma_{s}^{2}-\sigma_{T}^{2}=\sigma_{s}^{2}$ Eq. 11

In Supplementary Equation 10 we assumed that the volume fraction of sites available for C intercalation is $x$ < 1, because many sites are not available for C, due to the large surface to volume ratio. Therefore, the $1-x$ fraction of the volume has zero contribution to the static disorder, as that part of the volume does not change before, during and after the reaction. For the same reason, after the reaction (in the “off” state) the entire NP has only thermal disorder. A caveat: there is, of course, a non-zero static disorder in nanoparticles due to their surface tension effect and support interaction. But we assume that these effects do not change during the C intercalation and thus their respective contributions will cancel in Supplementary Equation 10. In the 4.4 nm NP (Supplementary Equation S11), we assume that $x\approx1$, because the surface to volume ratio is much smaller than in the 1.2 nm NP. By combining Supplementary Equations 10 and 11, and using experimental data shown in Supplementary Figure 28, we obtain the volume fraction of the C intercalation sites in the 1.2 nm NP:

$x=\frac{{\Delta\sigma}_{1.2nm}^{2}}{{\Delta\sigma}_{4.4nm}^{2}}$ Eq. 12

where the values for the numerator and the denominator are obtained from the experimental data shown in Supplementary Figure 28. Hence taking the volume fraction of C intercalation sites at the maximum obtained during the first ethene pulse for the two mean Ni particle sizes is (0.027-0.0208)/(0.0236-0.0155) = 0.76.

# Supplementary References

1. Goodman, D. W. Model Catalytic Studies over Metal Single Crystal. *Acc. Chem. Res.* **17**, 194–200 (1984).

2. Peyrovi, M. H. & Toosi, M. R. Study of benzene hydrogenation catalyzed by nickel supported on alumina in a fixed bed reactor. *React. Kinet. Catal. Lett.* **94**, 1–5 (2008).

3. Mattos, A. R. J. M., Probst, S. H., Afonso, J. C. & Schmal, M. Hydrogenation of 2-ethyl-hexen-2-al on Ni/Al_2_O_3_ catalysts. *J. Braz. Chem. Soc.* **15**, 760–766 (2004).

4. Nørskov, J. K. *et al.* The nature of the active site in heterogeneous metal catalysis. *Chem. Soc. Rev.* **37**, 2163–2171 (2008).

5. Martin, G. A. & Dalmon, J. A. Benzene hydrogenation over nickel catalysts at low and high temperatures: Structure-sensitivity and copper alloying effects. *J. Catal.* **75**, 233–242 (1982).

6. Andersson, M. P. *et al.* Structure sensitivity of the methanation reaction : H_2_-induced CO dissociation on nickel surfaces. *J. Catal.* **255**, 6–19 (2008).

7. Sehested, J., Dahl, S., Jacobsen, J. & Rostrup-Nielsen, J. R. Methanation of CO over nickel: mechanism and kinetics at high H_2_/CO ratios. *J. Phys. Chem. B* **109**, 2432–2438 (2005).

8. van Meerten, R. Z. C., Beaumont, A. H. G. M., van Nisselrooij, P. F. M. T. & Coenen, J. W. E. Structure sensitivity and crystallite size change of nickel during methanation of CO/H_2_ on nickel-silica catalysts. *Surf. Sci.* **135**, 565–579 (1983).

9. Vogt, C. *et al.* Unravelling structure sensitivity in CO_2_ hydrogenation over nickel. *Nat. Catal.* **1**, 127–134 (2018).

10. Vogt, C. *et al.* Understanding carbon dioxide activation and carbon-carbon coupling over nickel. *Nat. Commun.* (2018).

11. Iglesia, E. & Boudart, M. Structure-sensitivity and ensemble effects in reactions of strongly adsorbed intermediates. Catalytic dehydrogenation and dehydration of formic acid on nickel. *J. Phys. Chem.* **95**, 7011–7016 (1991).

12. Vogt, C., Kranenborg, J., Monai, M. & Weckhuysen, B. M. Structure Sensitivity in Steam and Dry Methane Reforming over Nickel: Activity and Carbon Formation. *ACS Catal.* **10**, 1428–1438 (2020).

13. Goodman, D. W. Ethane hydrogenolysis over single crystals of nickel: Direct detection of structure sensitivity. *Surf. Sci.* **123**, 679–685 (1982).

14. Hadj Romdhane, Y., Bellamy, B., De Gouveia, V., Masson, A. & Che, M. Structure sensitivity: hydrogenolysis of n-butane and hydrogenation of ethylene on nickel clusters condensed onto amorphous silica. *Appl. Surf. Sci.* **31**, 383–401 (1988).

15. Keane, M. A., Park, C. & Menini, C. Structure sensitivity in the hydrodechlorination of chlorobenzene over supported nickel. *Catal. Letters* **88**, 89–94 (2003).

16. Shu, Y., Lee, Y. K. & Oyama, S. T. Structure-sensitivity of hydrodesulfurization of 4,6- dimethyldibenzothiophene over silica-supported nickel phosphide catalysts. *J. Catal.* **236**, 112–121 (2005).

17. Keane, M. A. The liquid-phase hydrogenation of methylacetoacetate using nickel-exchanged Y zeolite catalysts: Part I. Structure sensitivity. *Zeolites* **13**, 14–21 (1993).

18. Mihaylov, M., Tsoncheva, T. & Hadjiivanov, K. Structure sensitivity of methanol decomposition on Ni/SiO_2_ catalysts. *J. Mater. Sci.* **46**, 7144–7151 (2011).

19. Zhang, J., Xu, H. & Li, W. Kinetic study of NH_3_ decomposition over Ni nanoparticles: The role of La promoter, structure sensitivity and compensation effect. *Appl. Catal. A Gen.* **296**, 257–267 (2005).

20. Guilleux, M. F., Dalmon, J. A. & Martin, G. A. Mechanism and structure sensitivity of propane hydrogenolysis over Ni/SiO2 catalysts. *J. Catal.* **62**, 235–242 (1980).

21. Koussathana, M., Vamvouka, D., Economou, H. & Verykios, X. Slurry-phase hydrogenation of aromatic compounds over supported noble metal catalysts. *Appl. Catal.* **77**, 283–301 (1991).

22. Kleis, J. *et al.* Finite size effects in chemical bonding: From small clusters to solids. *Catal. Letters* **141**, 1067–1071 (2011).

23. Crampton, A. S. *et al.* Structure sensitivity in the nonscalable regime explored via catalysed ethylene hydrogenation on supported platinum nanoclusters. *Nat. Commun.* **7**, 10389 (2016).

24. Kuo, C. Te, Lu, Y., Kovarik, L., Engelhard, M. & Karim, A. M. Structure Sensitivity of Acetylene Semi-Hydrogenation on Pt Single Atoms and Subnanometer Clusters. *ACS Catal.* **9**, 11030–11041 (2019).

25. Batteas, J., Dunphy, J., Somorjai, G. & Salmeron, M. Coadsorbate Induced Reconstruction of a Stepped Pt(111) Surface by Sulfur and CO: A Novel Surface Restructuring Mechanism Observed by Scanning Tunneling Microscopy. *Phys. Rev. Lett.* **77**, 534–537 (1996).

26. Marsh, A. L. & Somorjai, G. A. Structure, reactivity, and mobility of carbonaceous overlayers during olefin hydrogenation on platinum and rhodium single crystal surfaces. *Top. Catal.* **34**, 121–128 (2005).

27. Kieken, L. & Boudart, M. Rate of oxidation of CO on Pd at pressure between 10^-1^ and 10^2^ mbar. *Catal. Letters* **17**, 1–10 (1993).

28. Zaera, F. & Somorjai, G. A. Hydrogenation of Ethylene over Platinum (111) Single-Crystal Surfaces. *J. Am. Chem. Soc.* **106**, 2288–2293 (1984).

29. Bezemer, G. L. *et al.* Cobalt particle size effects in the Fischer-Tropsch reaction studied with carbon nanofiber supported catalysts. *J. Am. Chem. Soc.* **128**, 3956–3964 (2006).

30. a) Somorjai, G. A. The Catalytic Hydrogenation of Carbon Monoxide . The formation of C_1_ hydrocarbons the catalytic hydrogenation of carbon monoxide. The formation of C, hydrocarbons *Catal. Rev. Sci. Eng.* **4940**, 189–202 (1981).

b) Somorjai, G. A., Carrazza, J. Structure sensitivity of catalytic reaction. *Ind. Eng. Chem. Fundam.* **25**, 63-69 (1986).

31. Román-Martínez, M. C., Carzorla-Amorós, D., Salinas-Martínez de Lecea, C. & Linares-Solano, A. Structure sensitivity of CO_2_ hydrogenation reaction catalyzed by Pt/Carbon catalysts. *Langmuir* **12**, 379–385 (1996).

32. Wei, J. & Iglesia, E. Isotopic and kinetic assessment of the mechanism of methane reforming and decomposition reactions on supported Iridium catalysts. *Phys.Chem.Chem.Phys.* **6**, 3754–3759 (2004).

33. Wei, J. & Iglesia, E. Mechanism and site requirements for activation and chemical conversion of methane on supported Pt clusters and turnover rate comparisons among noble metals. *J. Phys. Chem. B* **108**, 4094–4103 (2004).

34. Spencer, N. D., Schoonmaker, R. C. & Somorjai, G. A. Iron single crystals as ammonia synthesis catalysts: surface structure on catalyst activity effect of. *J. Catal.* **135**, 129–135 (1982).

35. Meng, X. *et al.* Carbonylation of methanol to methyl acetate over Cu/TiO_2_-SiO_2_ catalysts: Influence of copper precursors. *Mol. Catal.* **456**, 1–9 (2018).

36. Huang, L. *et al.* Study on catalysis by carbonyl cluster-derived SiO_2_-supported rhodium for ethylene hydroformylation. *Catal. Letters* **32**, 61–81 (1995).

37. Hopstaken, M. J. P. & Niemantsverdriet, J. W. Structure sensitivity in the CO oxidation on rhodium: Effect of adsorbate coverages on oxidation kinetics on Rh(100) and Rh(111). *J. Chem. Phys.* **113**, 5457–5465 (2000).

38. Van Helden, P., Ciobica, I. M. & Coetzer, R. L. J. The size-dependent site composition of FCC cobalt nanocrystals. *Catal. Today* **261**, 48–59 (2016).

39. Baurecht, D. & Fringeli, U. P. Quantitative modulated excitation Fourier transform infrared spectroscopy. *Rev. Sci. Instrum.* **72**, 3782–3792 (2001).

40. Koh, H. P. & Hughes, R. Kinetics of ethylene hydrogenation over a supported nickel catalyst. *J. Catal.* **33**, 7–16 (1974).

41. Rideal, E. K. The hydrogenation of ethylene in contact with nickel. *J. Chem. Soc. Trans.* **121**, 309–318 (1922).

42. Pauls, A. C., Comings, E. W. & Smith, J. M. Kinetics of the hydrogenation of ethylene (on a nickel catalyst). *Canad. J. Chem.* **5**, 453–457 (1959).

43. Horiuti, J. & Miyahara, K. Hydrogenation of Ethylene on Metallic Catalysts (1934).

44. Godbey, D., Zaera, F., Yeates, R. & Somorjai, G. A. Hydrogenation of chemisorbed ethylene on clean, hydrogen, and ethylidyne covered platinum (111) crystal surfaces. *Surf. Sci.* **167**, 150–166 (1986).

45. Davis, S. M., Zaera, F., Gordon, B. E. & Somorjai, G. A. Radiotracer and thermal desorption studies of dehydrogenation and atmospheric hydrogenation of organic fragments obtained from [^14^C]ethylene chemisorbed over Pt(111) surfaces. *J. Catal.* **92**, 240–246 (1985).

46. Mohsin, S. B., Trenary, M. & Robota, H. J. Infrared Identification of the Low-Temperature Forms of Ethylene Adsorbed on platinum/alumina. *J. Phys. Chem.* **92**, 5229–5233 (1988).

47. Rekoske, J. & Cortright, R. Microkinetic analysis of diverse experimental data for ethylene hydrogenation on platinum. *J. Am. Chem. Soc.* **96**, 1880–1888 (1992).

48. Somorjai, G. A. New model catalysts (platinum nanoparticles) and new techniques (SFG and STM) for studies of reaction intermediates and surface restructuring at high pressures during catalytic reactions. *Appl. Surf. Sci.* **121**–**122**, 1–19 (1997).

49. Crampton, A. S. *et al.* Assessing the concept of structure sensitivity or insensitivity for sub-nanometer catalyst materials. *Surf. Sci.* **652**, 7–19 (2016).

50. Sabatier, P. & Senderens, J.-B. Hydrogénation directe des oxydes du carbone en présence de divers métaux divisés. *Compt. Rend.* **134**, 689–691 (1903).

51. Senderens, J.-B. & Sabatier, P. Nouvelles synthèses du méthane. *Compt. Rend.* **82**, 514–516 (1902).

52. Zhao, S. *et al.* Multimodal Study of the Speciations and Activities of Supported Pd Catalysts During the Hydrogenation of Ethylene. *J. Phys. Chem. C* **121**, 18962–18972 (2017).

53. Jung, U. *et al.* Comparative in operando studies in heterogeneous catalysis: Atomic and electronic structural features in the hydrogenation of ethylene over supported pd and pt catalysts. *ACS Catal.* **5**, 1539–1551 (2015).

54. Bugaev, A. L. *et al.* Palladium Carbide and Hydride Formation in the Bulk and at the Surface of Palladium Nanoparticles. *J. Phys. Chem. C* **122**, 12029–12037 (2018).

55. Li, L. *et al.* Noncrystalline-to-crystalline transformations in pt nanoparticles. *J. Am. Chem. Soc.* **135**, 13062–13072 (2013).

56. Dalba, G. & Fornasini, P. EXAFS Debye-Waller Factor and Thermal Vibrations of Crystals. *J. Synchrotron Rad.* **44**, 243–255 (1997).

57. Lander, J. J., Kern, H. E. & Beach, A. L. Solubility and diffusion coefficient of carbon in nickel: Reaction rates of nickel-carbon alloys with barium oxide. *J. Appl. Phys.* **23**, 1305–1309 (1952).

58. Siegel, D. J. & Hamilton, J. C. Computational study of carbon segregation and diffusion within a nickel grain boundary. *Acta Mater.* **53**, 87–96 (2005).
